# Integrative Multiomics and Single-Cell Profiling Identify TNFRSF1A^+^ Macrophages as a Prognostic and Therapeutic Target in Diffuse Large B-cell Lymphoma

**DOI:** 10.34133/csbj.0119

**Published:** 2026-06-01

**Authors:** Wenxian Yin, Xingyue Wang, Zhuo Chen, Mengqi Sun, Yinping Zhang, Jiahong Su, Zijun Yuan, Ting Xiao, Singkome Tima, Xi He, Zhangang Xiao, Suwit Duangmano

**Affiliations:** ^1^ Department of Medical Technology, Faculty of Associated Medical Sciences, Chiang Mai University, Chiang Mai 50200, Thailand.; ^2^ Department of Pharmacy, The Affiliated Traditional Chinese Medicine Hospital, Southwest Medical University, Luzhou 646000, China.; ^3^ Laboratory of Molecular Pharmacology, Department of Pharmacology, School of Pharmacy, Southwest Medical University, Luzhou 646000, China.; ^4^ Infectious Disease Department, The Six School of Clinical Medicine, the Affiliated Qingyuan Hospital (Qingyuan People’s Hospital), Guangzhou Medical University, Guangzhou 511518, China.; ^5^ Drug Clinical Trial Institution, the Affiliated Hospital, Southwest Medical University, Luzhou 646000, China.; ^6^ Cell Therapy & Cell Drugs of Luzhou Key Laboratory, Luzhou 646000, China.; ^7^ South Sichuan Institute of Translational Medicine, Luzhou 646000, China.

## Abstract

The tumor microenvironment plays a critical role in therapeutic resistance in diffuse large B-cell lymphoma (DLBCL), yet actionable targets within the immune cells remain largely undefined. Through integrative multiomics profiling, we identified tumor necrosis factor receptor superfamily member 1A (*TNFRSF1A*) as a key prognostic determinant associated with predicted R-CHOP (rituximab, cyclophosphamide, doxorubicin, vincristine, and prednisone) resistance, specifically localizing its expression to tumor-associated macrophages (TAMs). Mechanistic investigation via ligand–receptor network analysis and in silico perturbation revealed that TNFRSF1A^+^ TAMs sustain a pro-oncogenic and immunosuppressive niche, by driving nuclear factor-κB (NF-κB)-dependent B-cell activating factor (BAFF) secretion and immune checkpoint ligand (programmed death-ligand 1/programmed death-ligand 2) expression. Pharmacogenomic screening coupled with molecular dynamics simulations identified curcumin as a potential modulator of TNFRSF1A-associated signaling. Subsequent experimental validation using small interfering RNA-mediated knockdown and coculture assays confirmed that disrupting the macrophage-intrinsic TNFRSF1A/NF-κB/BAFF axis eliminated the protective support provided by TAMs to lymphoma cells. Additionally, a novel risk model derived from the TNFRSF1A^+^ TAM transcriptional signature demonstrated robust predictive accuracy for patient outcomes across independent cohorts. Our findings elucidate the role of TNFRSF1A^+^ TAMs in driving DLBCL progression, highlight the TNFRSF1A/NF-κB/BAFF axis as a vulnerability in the DLBCL microenvironment, and propose curcumin as a viable therapeutic strategy to disrupt this supportive microenvironment.

## Introduction

Diffuse large B-cell lymphoma (DLBCL) is the most common aggressive subtype of B-cell lymphoma and a leading cause of lymphoma-related morbidity and mortality globally [1]. Despite the established efficacy of R-CHOP (rituximab, cyclophosphamide, doxorubicin, vincristine, and prednisone) in a majority of patients, nearly 40% succumb to primary refractory disease or relapse, reflecting substantial clinical and biological heterogeneity [2]. This heterogeneity, which encompasses divergent mutational landscapes (e.g., *MYD88*, *CD79B*, and *CARD11*) and variable dependence on oncogenic pathways such as nuclear factor-κB (NF-κB), makes it difficult to predict clinical outcomes and to design uniformly effective targeted interventions [3,4]. Increasing evidence suggests that tumor-intrinsic alterations alone cannot fully account for treatment failure, highlighting the tumor microenvironment (TME) as a pivotal driver of disease progression and therapeutic resistance in DLBCL [5].

Tumor-associated macrophages (TAMs) are among the most abundant and functionally diverse immune populations within the DLBCL TME. While the prognostic importance of TAMs, particularly those exhibiting M2-like signatures, has been well documented [6], recent advances in single-cell and spatial transcriptomics have provided a higher-resolution view of this complexity. Distinct TAM subsets interact with malignant B cells and other immune components, modulating immune suppression, tumor cell survival, and treatment response [7]. At the molecular level, NF-κB signaling within macrophages plays a central role in regulating cytokine secretion, immune checkpoint ligand expression, and survival programs; however, the mechanisms that sustain NF-κB-dependent protumor functions in DLBCL TAMs remain incompletely defined [8]. Advances in integrative multiomics profiling have enabled systematic characterization of immune heterogeneity and ligand–receptor interactions within the lymphoma TME [9]. Prior studies have implicated TAM-derived survival factors, such as B-cell activating factor (BAFF), in supporting malignant B-cell fitness and have highlighted macrophage expression of immune checkpoint ligands, such as programmed death-ligand 1 (PD-L1), as a contributor to local immune evasion [10,11]. Nevertheless, it remains unclear whether a macrophage-intrinsic factor can simultaneously identify a prognostically unfavorable TAM population, mechanistically link NF-κB-driven BAFF and immune checkpoint programs, and serve as a viable target for therapeutic intervention.

Tumor necrosis factor receptor superfamily member 1A (*TNFRSF1A*, also known as *TNFR1*) is a promising candidate for mediating these processes. TNFRSF1A functions as a canonical signaling receptor that links extracellular inflammatory stimuli to downstream NF-κB activation through the assembly of signaling complexes, thereby regulating macrophage polarization and inflammatory responses [12]. While TNFRSF1A signaling has been well characterized in malignant cells, its selective enrichment, functional role, and clinical relevance within TAM subsets in DLBCL have not been systematically investigated. Moreover, although natural compounds such as curcumin have demonstrated immunomodulatory and anti-lymphoma activities, the receptor-mediated mechanisms underlying their effects on TAM subsets remain poorly understood [13].

**Fig. 1.**
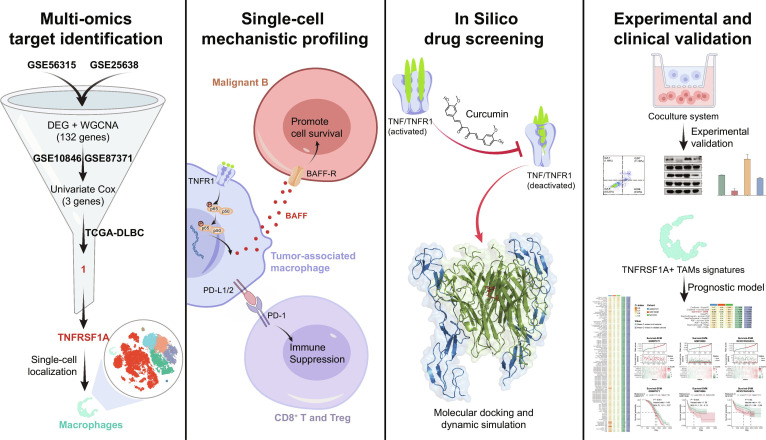
Schematic overview of the study design and analytical workflow.

In this study, we integrate large-scale bulk transcriptomic datasets with single-cell and spatial analyses to elucidate the role of *TNFRSF1A* in DLBCL (Fig. 1). We identified TNFRSF1A as a marker of a distinct TAM subset associated with adverse prognosis and predicted R-CHOP resistance. Mechanistically, through genetic perturbation and ligand–receptor modeling, we show that TNFRSF1A^+^ TAMs maintain a protumoral and immunosuppressive microenvironment via NF-κB-dependent BAFF secretion and PD-L1 expression. Furthermore, we identify curcumin as a potential modulator of TNFRSF1A-associated signaling that disrupts this supportive microenvironment. Taken together, these findings identify TNFRSF1A^+^ TAMs as a mechanistically defined and clinically relevant immunoregulatory population in DLBCL.

## Materials and Methods

### Data acquisition and preprocessing

Six bulk transcriptomic datasets (GSE56315 [14], GSE25638 [15], GSE10846 [16], GSE87371 [17], The Cancer Genome Atlas [TCGA]-diffuse large B-cell lymphoma [DLBC], and National Cancer Institute Center for Cancer Research [NCICCR]-DLBCL) were obtained from Gene Expression Omnibus, TCGA, and Genomic Data Commons databases. For the discovery phase, raw data from GSE56315 and GSE25638 were merged, and batch-effect correction was performed using the “ComBat” algorithm in the sva R package [18]. Differentially expressed genes between tumor and normal tissues were identified using the limma R package [19] with |*log*_*2*_*FC*| > 1 and *p.adj* < 0.05. Single-cell RNA sequencing (scRNA-seq) data from GSE182434 [20] and VRJUNV [21] were processed using the Seurat R package [22]. Stringent quality control was applied to retain high-quality cells (200 < *nFeature_RNA* < 6,000, 500 < *nCount_RNA* < 60,000, and *percent.mt* < 15%). Doublets were predicted and excluded using the DoubletFinder R package [23]. To integrate datasets and eliminate batch effects, canonical correlation analysis was employed. Following normalization and principal component analysis on highly variable genes, the top 30 principal components were selected for unsupervised clustering with a resolution of 0.6. Cell clusters were visualized using t-distributed stochastic neighbor embedding and annotated based on canonical marker expression: B cells (*CD79A*, *CD79B*, and *MS4A1*), T cells (*CD3D*, *CD3E*, and *CD3G*), naive T cells (*CCR7*, *TCF7*, and *LEF1*), CD8^+^ T cells (*CD8A*, *GZMK*, and *CCL5*), CD4^+^ T cells (*CD4*, *CD40LG*, and *FOXP3*), macrophages (*CD68*, *FCGR3A*, and *CD163*), natural killer cells (*GNLY*, *KLRD1*, and *NKG7*), and dendritic cells (DCs) (*ITM2C*, *LILRA4*, and *IL3RA*). For downstream comparative and intercellular communication analyses, macrophages were further stratified into TNFRSF1A^+^ and TNFRSF1A^−^ subsets. Specifically, cells with a normalized expression count of *TNFRSF1A* > 0 were defined as TNFRSF1A^+^ TAMs, while cells with zero detected counts were classified as TNFRSF1A^−^ TAMs. Additionally, the 6 spatially derived macrophage signatures (MacroSigs) from GSE232853 [7] were directly retrieved from the original study for downstream analysis.

### WGCNA and identification of prognostic candidates

Coexpression network analysis was performed using the Weighted Gene Co-expression Network Analysis (WGCNA) R package [24] on the merged bulk RNA-seq cohort. A power of 12 was selected as the soft-thresholding power to satisfy a scale-free network topology. Coexpression modules were identified via average linkage hierarchical clustering based on a topological overlap matrix-based dissimilarity measure and the dynamic tree-cut algorithm. Module–trait relationships were evaluated by correlating module eigengenes with clinical features, specifically selecting modules exhibiting the highest correlation with the tumor phenotype. The key module genes were intersected with the identified differentially expressed genes to identify candidate genes. To assess the prognostic value of these genes, univariate Cox regression analysis was conducted for each intersected gene. Candidates with a hazard ratio (*HR*) > 1 and *P* < 0.05 were identified as significant prognostic factors.

### Inference of malignancy and intercellular communication analysis

To distinguish malignant B cells from normal counterparts, large-scale chromosomal copy number variations (CNVs) were estimated using the inferCNV R package (https://github.com/broadinstitute/inferCNV), utilizing B cells from reactive lymph nodes (rLNs) as the normal reference. Intercellular communication networks within the TME were modeled using the CellChat R package [25]. The interaction strength and probability between distinct cell populations were computed based on the expression of known ligand–receptor pairs using the CellChatDB.human database.

### Functional enrichment analysis

To characterize biological functions and signaling pathways associated with *TNFRSF1A* expression, functional enrichment analyses were performed using the clusterProfiler R package [26]. Reference gene sets, including the Hallmark and Kyoto Encyclopedia of Genes and Genomes pathways, were obtained via the msigdbr R package [27]. Gene set enrichment analysis (GSEA) was performed to evaluate pathway activity differences between defined groups.

### Virtual knockout analysis

To evaluate the regulatory impact *TNFRSF1A* depletion on macrophage transcriptional networks, in silico knockout analysis was conducted using the scTenifoldKnk R package [28]. First, a wild-type single-cell gene regulatory network was constructed from the normalized expression matrix of TAMs. A “pseudo-knockout (KO)” network was generated by virtually removing the target gene (*TNFRSF1A*) and its associated edges. Manifold alignment was employed to compare the topological differences between the WT and knockout networks. Genes exhibiting significant perturbation following the virtual knockout were identified based on a threshold of |*z-score*| > 1.5.

### Immune infiltration estimation and pharmacogenomic screening

The relative abundance of infiltrating immune cells in bulk transcriptomic samples was quantified using the CIBERSORT R package [29]. The association between *TNFRSF1A* expression and the estimated proportions of specific immune cell populations was evaluated using Spearman correlation analysis.

To predict therapeutic drug response, pharmacogenomic screening was conducted using the oncoPredict R package [30]. Using the Genomics of Drug Sensitivity in Cancer (GDSC1) database as the training set, a ridge regression model was constructed to estimate the half-maximal inhibitory concentration (*IC*_*50*_) of chemotherapeutic agents for each bulk transcriptomic sample.

### Molecular docking and dynamics simulation

Molecular docking was performed using LeDock (v1.0) to investigate curcumin-target binding interactions [31]. The ligand structure (Compound Identifier [CID]: 969516) was downloaded from PubChem (https://pubchem.ncbi.nlm.nih.gov/) and energy minimized in Chem3D 22.0 with MMFF94 force field. Protein crystal structures (Protein Data Bank [PDB] ID: 7kpb) were acquired from RCSB PDB (https://www.rcsb.org/) under stringent criteria: (a) x-ray diffraction resolution ≤ 3.0 Å, (b) *Homo sapiens* origin, and (c) ligand-bound conformations. Receptor preparation involved adding hydrogen atoms, assigning partial charges, and removing crystallographic water molecules. Docking parameters were optimized with a grid spacing of 0.4 Å and an energy scoring cutoff of −8 kcal/mol. Interaction analysis and visualization was conducted in Discovery Studio (v4.5).

Molecular dynamics (MD) simulations were executed in GROMACS (v2020.6) using the Amber14SB force field. The ligand topology was generated via ACPYPE, while protein parameters were prepared using the gmx pdb2gmx module. The system was solvated in TIP3P water with 1.2-nm periodic boundary distance and neutralized by adding Na^+^ and Cl^−^ ions to a concentration of 0.15 M. Energy minimization (5,000 steps), followed by NVT (100 ps) and NPT (100 ps) equilibration phases. Production MD ran for 100 ns with 2-fs timestep under LINear Constraint Solver (LINCS) constraints. Trajectory analysis included root-mean-square deviation (RMSD), root-mean-square fluctuation (RMSF), radius of gyration, and hydrogen bond calculations using the built-in GROMACS analysis tools (gmx rms, rmsf, gyrate, sasa, and hbond). Gibbs free energy was estimated via MM/PBSA (v16.0) with the igb = 2 generalized Born solvent model [32]. Data visualization was performed using DuIvyTools (v0.6.0).

### Construction and validation of prognostic model

The prognostic model was developed using the Mime R package [33], which incorporates various machine learning frameworks for survival analysis. Marker genes of TNFRSF1A^+^ TAMs identified from scRNA-seq were used as input features. To prevent overfitting and ensure generalizability, a 10-fold cross-validation strategy was implemented within the training cohort. A total of 101 combinations of machine learning algorithms were evaluated. Feature selection and model optimization were driven by maximizing the concordance index (C-index) and minimizing the performance gap between the training set (GSE87371) and the external validation cohorts (GSE10846 and NCICCR-DLBCL). These validation cohorts were not used in model training or parameter tuning. The optimal model was selected based on its stable prognostic performance across all cohorts. The risk score for each patient was then calculated, and the model’s predictive performance was validated using Kaplan–Meier survival analysis, time-dependent receiver operating characteristic curves, calibration plots, decision curve analysis, and univariate Cox regression.

### Cell culture and coculture system

The human DLBCL cell line WSU-DLCL2 (Cat#: CTCC-003-0027, Zhejiang Meisen Cell Technology Co., Ltd., Jinhua, China) and the human monocyte/macrophage cell line THP-1 (Cat#: CX0042, Wuhan Boster Biological Technology Co., Ltd., Wuhan, China) were maintained in RPMI 1640 medium (Procell Life Science & Technology Co., Ltd., Wuhan, China) supplemented with 10% fetal bovine serum (Shanghai XP Biomed Ltd., Shanghai, China) and 1% penicillin/streptomycin (Beyotime Biotech Inc., Shanghai, China) at 37 °C with 5% CO_2_ in a humidified atmosphere. THP-1 cells (5 × 10^5^ cells/well) were seeded in 6-well plates and treated with 100 ng/ml phorbol 12-myristate 13-acetate (Cat#: TQ0198, TargetMol Chemicals Inc., Boston, USA) for 24 h, followed by culture in fresh medium for an additional 24 h to obtain M0 macrophages (M-THP1). For coculture experiments, a Transwell system (0.4-μm pore size) (Corning Inc., New York, USA) was utilized to simulate the tumor cell–macrophage crosstalk. WSU-DLCL2 cells (lower chamber, 3 × 10^5^ cells/well) were cocultured with M-THP1 cells (upper chamber, 1 × 10^5^ cells/well) for 48 h.

### siRNA transfection

Small interfering RNAs (siRNAs) targeting *TNFRSF1A* (si-*TNFRSF1A*) and a nontargeting negative control (si-NC) were designed and synthesized by Tsingke Biotechnology Co., Ltd. (Beijing, China). M-THP1 cells were seeded in 6-well plates and transfected at approximately 70% confluence. Transfection complexes were prepared by diluting 2 μl of siRNA stock solution (20 μM) and 4 μl of jetPRIME transfection reagent (Polyplus-transfection, Illkirch, France) into 200 μl of jetPRIME buffer. After a 10-min incubation at room temperature, the mixtures were added to the cells in fresh complete medium. At 24 h posttransfection, the cells were harvested and reseeded for subsequent functional coculture assays. At 72 h posttransfection, cell pellets were collected for Western blot analysis to verify knockdown efficiency. The siRNA sequences used in this study were as follows: si-TNFRSF1A #1: 5'-GAGCUUGAAGGAACUACUA-3'; si-TNFRSF1A #2: 5'-GCUGCAGGAAGAACCAGUA-3'; si-TNFRSF1A #3: 5'-CUGUAGUAACUGUAAGAAA-3'.

### Curcumin treatment

Curcumin (Cat#: T1516, 98.98%, TargetMol Chemicals Inc., Boston, USA) was dissolved in dimethyl sulfoxide (Merck KGaA, Darmstadt, Germany) to prepare a 10 mM stock solution. Complete medium was used to serially dilute the stock solution to yield final concentrations of 1, 2, 4, 8, 16, 32, 64, and 128 μM. Cell viability was evaluated using the Cell Counting Kit-8 assay (APExBIO Tech Co., Ltd., Houston, USA) after 24, 48, and 72 h of treatment. Based on the concentration–time response curves, appropriate concentration and time were selected for subsequent experiments. Curcumin-containing medium was added to the macrophage culture medium immediately after initiating coculture.

### Cell proliferation and apoptosis analysis

After 48 h, tumor cell concentration in the lower chamber was determined using a hemocytometer and an inverted microscope. For apoptosis assessment, cells were harvested, washed with phosphate-buffered saline, and stained with Annexin V-fluorescein isothiocyanate and propidium iodide (BD Biosciences, Franklin Lakes, USA) according to the manufacturer’s protocol. The percentage of apoptotic cells was determined using flow cytometry (BD FACSCalibur, Franklin Lakes, USA), and data were analyzed with FlowJo software (v10.8.1).

### Reverse transcription quantitative polymerase chain reaction

Total RNA was extracted using TRIzol reagent (Thermo Fisher Scientific Inc., Waltham, USA) following the manufacturer’s protocol. Reverse transcription was performed with HiScript III qRT SuperMix (Nanjing Vazyme Biotech Co., Ltd., Nanjing, China) following the manufacturer’s instructions. The synthesized cDNA was diluted 5-fold with nuclease-free water and stored at −20 °C. Quantitative polymerase chain reaction (qPCR) was performed using ChamQ Universal SYBR qPCR Master Mix (Nanjing Vazyme Biotech Co., Ltd., Nanjing, China) on a CFX96 Real-Time PCR Detection System (Bio-Rad Laboratories, Inc., Hercules, USA). Gene-specific primers (Table S1) were designed and synthesized by Sangon Biotech Co., Ltd. (Shanghai, China). Each reaction included 3 technical replicates, and data were normalized to glyceraldehyde 3-phosphate dehydrogenase and analyzed via the 2^−ΔΔCt^ method.

### Western blot analysis

Total protein was extracted using radioimmunoprecipitation assay lysis buffer (Beyotime Biotech Inc., Shanghai, China) containing protease/phosphatase inhibitors (F. Hoffmann-La Roche Ltd., Basel, Switzerland). Protein concentrations were quantified using a bicinchoninic acid protein assay kit (Pierce Manufacturing Inc., Appleton, USA). Equal amounts (30 μg) of protein were separated via 10% sodium dodecyl sulfate polyacrylamide gel electrophoresis and electro-transferred to polyvinylidene fluoride membranes (MilliporeSigma, Massachusetts, USA), and blocked with 5% nonfat milk. Membranes were incubated overnight at 4 °C with primary antibodies: TNFR1 (1:1,000, Cat#: 21574-1-AP) was purchased from Proteintech Group, Inc., Illinois, USA; p-p65 (1:1,000, Cat#: F0155), p-IκBα (1:1,000, Cat#: A5983), p65 (1:1,000, Cat#: F0006), IκBα (1:1,000, Cat#: A5599), and β-actin (1:5,000, Cat#: F0012) were purchased from Selleck Chemicals LLC, Houston, USA. Horseradish peroxidase-conjugated secondary antibodies (1:5,000, Cat#: 7074S, Cell Signaling Technology, Inc., Danvers, USA) were applied for 1 h at room temperature. Protein bands were detected using enhanced chemiluminescence substrate (Bio-Rad Laboratories, Inc., Hercules, USA) and quantified by ImageLab software (v6.0).

### Enzyme-linked immunosorbent assay (ELISA)

Cell-free supernatants were collected from the upper chamber of the Transwell system after 48 h of coculture. The supernatant was centrifuged at 1,000 × g for 10 min (4 °C) and filtered through a 0.22-μm sterile membrane to remove cellular debris. Secreted BAFF levels were quantified using a Human BAFF/TNFSF13B ELISA Kit (Cat#: 64907H1, Wuhan Meimian Tech Co., Ltd., Wuhan, China) according to the manufacturer’s protocol.

### Statistical analysis

Statistical analyses and data visualization were performed using R software (v4.3.0) and GraphPad Prism (v9.5.1). For bioinformatics analyses, differential expression was adjusted using the Benjamini–Hochberg method to control the false discovery rate. Correlation coefficients were calculated using the Spearman method, and survival differences were determined via the Kaplan–Meier method with the log-rank test. For experimental data, the normality of distribution was assessed using the Shapiro–Wilk test. Comparisons between 2 groups were performed using the unpaired Student *t* test (for normally distributed data) or the Mann–Whitney U test for non-normally distributed data. Multigroup comparisons were conducted using 1-way analysis of covariance (ANOVA) followed by Tukey’s post hoc test or the Kruskal–Wallis test. Statistical significance was defined as *P* < 0.05 (^*^P < 0.05, ^**^P < 0.01, ^***^P < 0.001, ^****^P < 0.0001; ns, not significant).

## Results

### Identification of *TNFRSF1A* as a potential prognostic biomarker associated with R-CHOP resistance in DLBCL

**Fig. 2.**
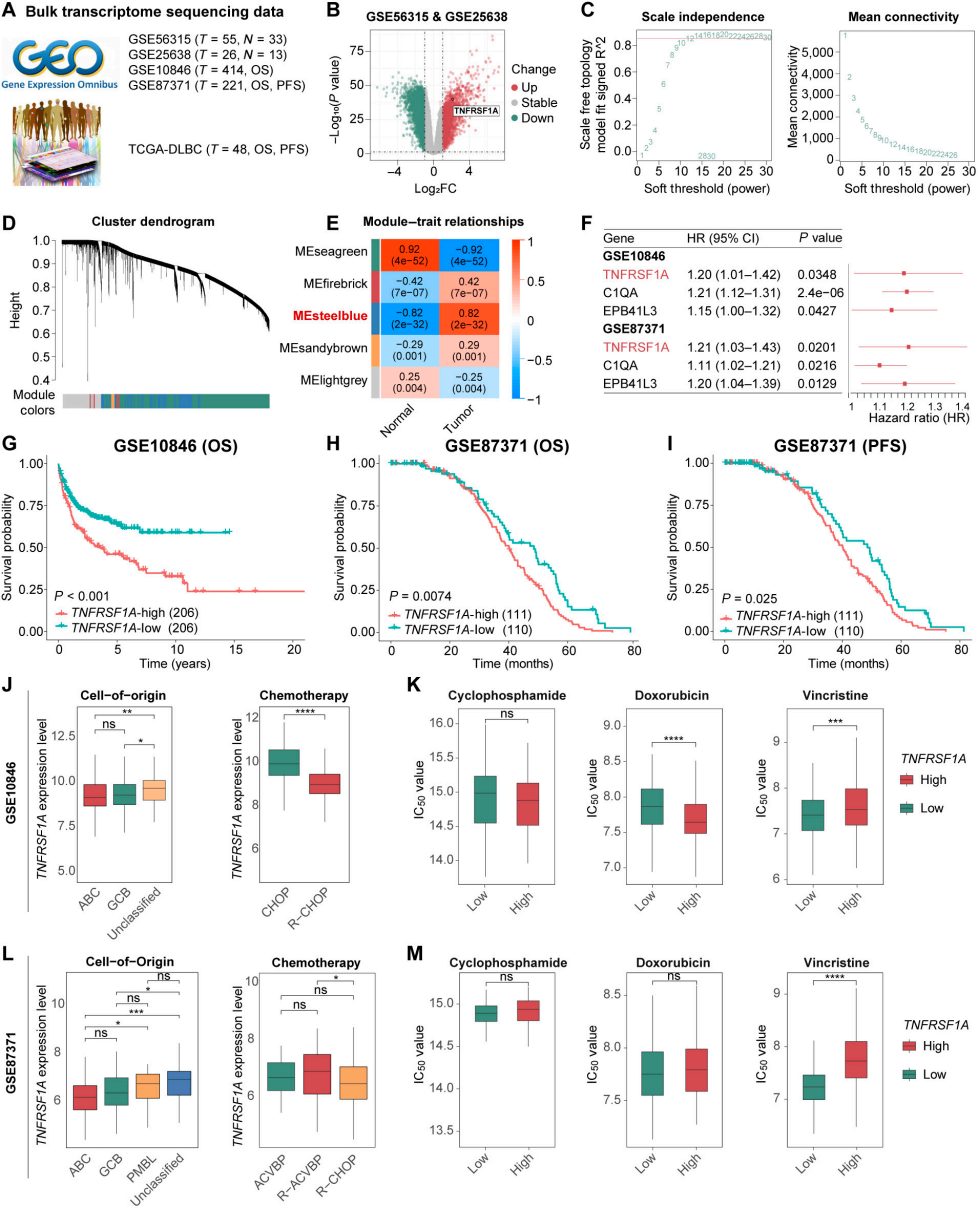
Multiomics screening identifies tumor necrosis factor receptor superfamily member 1A (*TNFRSF1A*) as a potential therapeutic target in diffuse large B-cell lymphoma (DLBCL). (A) Summary of bulk transcriptomic datasets utilized for discovery and validation. (B) Volcano plot of differentially expressed genes (DEGs) between DLBCL and normal tissues in the merged discovery cohort. (C to E) Weighted Gene Co-expression Network Analysis (WGCNA): (C) soft-thresholding power selection (*β* = 12) and mean connectivity analysis, (D) hierarchical clustering of coexpression modules, and (E) module–trait correlation heatmap highlighting the steelblue module. (F) Forest plot of univariate Cox regression for candidate genes across validation cohorts. (G to I) Kaplan–Meier analysis of overall survival (OS) (G and H) and progression-free survival (PFS) (I) in *TNFRSF1A*-high versus -low groups. (J and L) *TNFRSF1A* expression across cell-of-origin (COO) subtypes and chemotherapy regimens in GSE10846 (J) and GSE87371 (L). (K and M) Estimated half-maximal inhibitory concentration (*IC*_*50*_) values for cyclophosphamide, doxorubicin, and vincristine using the oncoPredict algorithm in validation cohorts. *P < 0.05, **P < 0.01, ***P < 0.001, ****P < 0.0001; ns, not significant.

To identify robust prognostic biomarkers in DLBCL, we performed an integrative analysis utilizing 5 independent transcriptomic cohorts (GSE56315, GSE25638, GSE10846, GSE87371, and TCGA-DLBC) (Fig. 2A). Initially, differential expression analysis on the batch-corrected merged dataset (GSE56315 and GSE25638) identified 1,842 up-regulated and 3,303 down-regulated genes using a threshold of |*log*_*2*_*FC*| > 1 and *p.adj* < 0.05 (Fig. 2B and Fig. S1A to F). In parallel, WGCNA highlighted the “steelblue” module as being positively correlated with the tumor phenotype (*r* = 0.82, *P* = 2 × 10^−32^) (Fig. 2C to E). Intersection of the module membership with the up-regulated gene signature yielded 132 candidate genes associated with DLBCL progression (Table S2). To identify targets with prognostic significance, we subjected these candidates to univariate Cox regression analysis within the GSE10846 and GSE87371 cohorts, which provided complete survival information. Among these, *TNFRSF1A*, *C1QA*, and *EPB41L3* consistently emerged as significant risk factors across both datasets (*HR* > 1, *P* < 0.05) (Fig. 2F). While all 3 candidates held prognostic value, *TNFRSF1A* was prioritized for downstream investigation due to its superior druggability and mechanistic relevance. Unlike the secreted protein *C1QA* or the intracellular protein *EPB41L3*, *TNFRSF1A* encodes a transmembrane receptor, making it a high-priority target for therapeutic modulation. Furthermore, *TNFRSF1A* acts as a key upstream activator of the canonical NF-κB pathway—a central driver of DLBCL progression—and its consistent overexpression was further confirmed in the TCGA-DLBC cohort (Fig. S1G).

Translating molecular discoveries into clinically relevant biomarkers is a fundamental goal of precision oncology [33]. To assess the clinical relevance of *TNFRSF1A* and its role in therapeutic response, we systematically analyzed *TNFRSF1A* expression in the GSE10846 and GSE87371 cohorts across gender, age, treatment regimen, disease stage, and cell-of-origin (COO) subtypes (Fig. S2A and B). Kaplan–Meier survival analyses indicated that patients with high *TNFRSF1A* expression exhibited significantly inferior overall survival across both datasets (Fig. 2G and H) and shorter progression-free survival in the GSE87371 cohort (Fig. 2I), a trend consistently observed in the TCGA cohort (Fig. S1H and I). Within this framework, *TNFRSF1A* expression was strongly associated with COO classification, showing preferential enrichment in Unclassified/primary mediastinal B-cell lymphoma subtypes compared with the germinal center B-cell-like/activated B-cell-like (ABC) subtypes, whereas no significant associations were observed with age, gender, International Prognostic Index, or stage. Notably, analysis of treatment regimens revealed that *TNFRSF1A* expression levels varied significantly among patients undergoing standard R-CHOP or R-ACVBP (rituximab, doxorubicin, cyclophosphamide, vindesine, bleomycin, and prednisone) immunochemotherapy, suggesting a potential association with predicted therapeutic response (Fig. 2J and L and Fig. S2C and D). To further investigate its prognostic impact across different molecular contexts, we performed subgroup analyses based on COO classification (Fig. S3). High *TNFRSF1A* expression significantly predicted poor survival in the Unclassified subtype (*P* = 0.013, GSE10846), while consistent trends toward inferior outcomes were observed in the ABC and germinal center B-cell-like subtypes, despite limited sample sizes in these specific strata.

Based on these findings, we evaluated the pharmacogenomic landscape using the oncoPredict algorithm to assess sensitivity to R-CHOP components. Specifically, elevated *TNFRSF1A* levels were consistently associated with higher predicted *IC*_*50*_ values for vincristine in both independent cohorts, whereas predicted responses to cyclophosphamide and doxorubicin were variable (Fig. 2K and M). These algorithm-based predictions suggest that *TNFRSF1A* overexpression is linked to reduced sensitivity to specific R-CHOP components, particularly vincristine.

### Single-cell profiling reveals that *TNFRSF1A* is predominantly enriched in TAMs

**Fig. 3.**
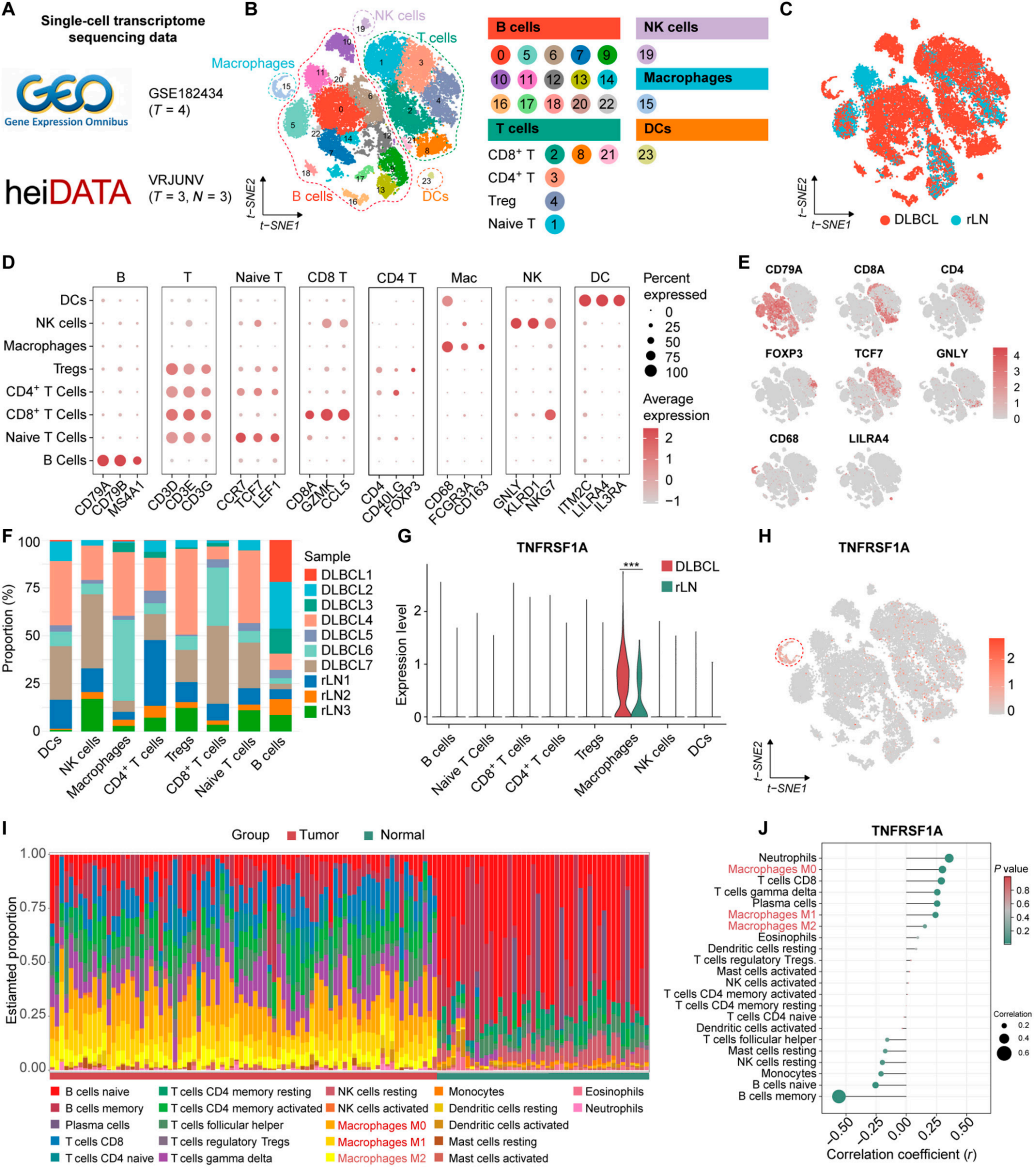
Preferential expression of tumor necrosis factor receptor superfamily member 1A (*TNFRSF1A*) in tumor-associated macrophages (TAMs) of diffuse large B-cell lymphoma (DLBCL). (A) Summary of single-cell RNA sequencing (scRNA-seq) datasets used in the study. (B and C) t-distributed stochastic neighbor embedding (t-SNE) visualization of integrated single-cell transcriptomes stratified by (B) cell types and (C) sample origin (DLBCL versus reactive lymph node [rLN]). (D and E) Validation of cell identities through (D) dot plot of canonical markers and (E) feature plots of lineage-specific markers. (F) Relative cell-type proportions across individual samples. (G and H) Macrophage-specific enrichment of *TNFRSF1A* visualized by (G) violin plot and (H) feature plot. (I) Comparative analysis of estimated immune cell abundance between tumor and normal tissues in bulk transcriptomic cohorts. (J) Correlation between *TNFRSF1A* expression and infiltrating immune cell proportions, highlighting a robust association with macrophages. ***P < 0.001.

scRNA-seq has revolutionized our ability to dissect cellular heterogeneity and spatial organization within the TME [34]. To spatially resolve the expression of *TNFRSF1A* within the DLBCL TME, we performed an integrative analysis of single-cell transcriptomic data from the GSE182434 and heiDATA-VRJUNV datasets (Fig. 3A). After stringent quality control, batch effects were effectively removed using canonical correlation analysis, enabling the identification of 5 major cell types via t-distributed stochastic neighbor embedding clustering (Fig. 3B and C and Fig. S4A to D and F).

Cell identities, including B cells, T cell subsets (CD4^+^, CD8^+^, naive, and regulatory T), natural killer cells, macrophages, and DCs, were rigorously validated by both canonical marker expression and functional pathway enrichment analysis of lineage-specific signatures (Fig. 3D and E and Fig. S4E and G). Compositional analysis further revealed substantial interpatient heterogeneity within the TME (Fig. 3F). Specifically, evaluation of *TNFRSF1A* expression revealed a significant preferential enrichment within the macrophage cluster compared to other cell types. Moreover, differential expression analysis confirmed that *TNFRSF1A* levels were significantly up-regulated in TAMs from DLBCL tissues relative to macrophages from rLNs (Fig. 3G and H). These single-cell observations were confirmed in bulk transcriptomic cohorts via immune infiltration algorithms, which demonstrated expanded macrophage infiltration in tumor tissues and identified a robust positive correlation between *TNFRSF1A* expression and macrophage abundance, encompassing various polarization states (Fig. 3I and J). Collectively, these multiomics data establish *TNFRSF1A* as a predominant marker of TAMs in DLBCL.

### TNFRSF1A^+^ TAMs foster a prosurvival and immunosuppressive microenvironment via NF-κB-mediated BAFF secretion and immune checkpoint molecule expression

**Fig. 4.**
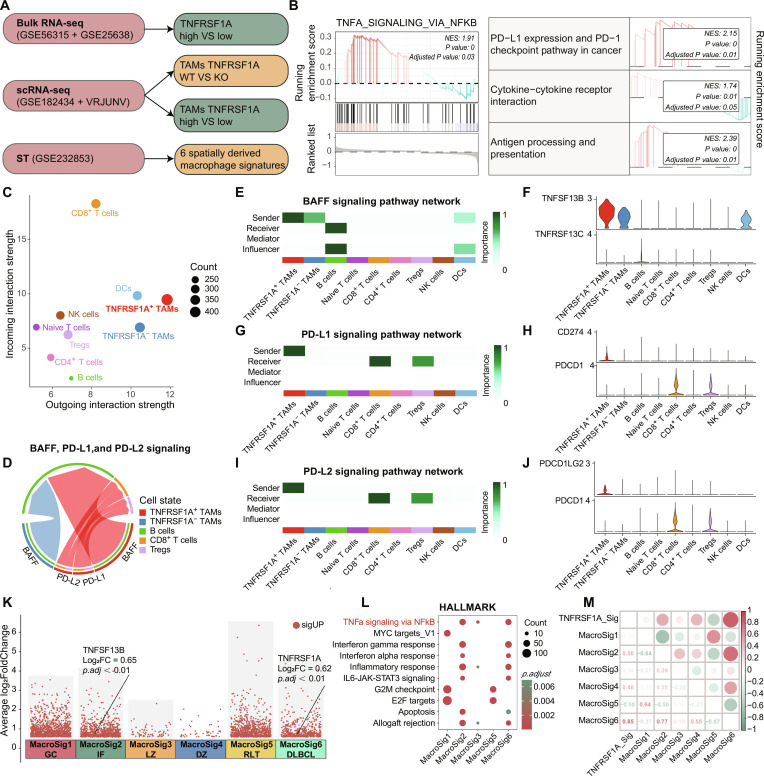
Tumor necrosis factor receptor superfamily member 1A-positive (TNFRSF1A^+^) tumor-associated macrophages (TAMs) drive diffuse large B-cell lymphoma (DLBCL) progression via B-cell activating factor (BAFF) and programmed death-ligand 1 (PD-L1) signaling. (A) Multiomics integration workflow for mechanistic dissection of TNFRSF1A-mediated signaling. (B) Gene set enrichment analysis (GSEA) of single-cell RNA sequencing (scRNA-seq) data highlighting activation of tumor necrosis factor (TNF)/nuclear factor-κB (NF-κB) and checkpoint pathways in TNFRSF1A^+^ TAMs. (C) Interaction strength analysis identifying TNFRSF1A^+^ TAMs as primary signal senders. (D to J) CellChat-based intercellular communication: (D) chord diagram of dominant ligand–receptor axes, and signaling probabilities/expression profiles of (E and F) BAFF, (G and H) PD-L1, and (I and J) PD-L2 pathways. (K to M) Spatial transcriptomic validation: (K) Volcano plot, (L) pathway enrichment across 6 spatial macrophage signatures (MacroSigs), and (M) correlation between scRNA-seq-derived TNFRSF1A_Sig and spatial transcriptomics (ST)-defined MacroSigs.

To elucidate the molecular mechanisms by which TNFRSF1A drives DLBCL progression, we integrated bulk RNA-seq, scRNA-seq, and spatial transcriptomics data (Fig. 4A). We first validated the malignant identity of B cells in DLBCL samples using inferCNV (Fig. S5A). Based on this result, subsequent cell–cell communication analyses were restricted to the DLBCL group. Macrophages were stratified into TNFRSF1A^+^ and TNFRSF1A^−^ subsets according to *TNFRSF1A* expression (Fig. S5C and D). Interaction strength analysis identified TNFRSF1A^+^ TAMs as a major source of outgoing signals, while CD8^+^ T cells constituted the dominant recipients within the communication network (Fig. 4C and Fig. S5E). Ligand–receptor pair analysis and pathway modeling identified TNFRSF1A^+^ TAMs as key mediators for BAFF, PD-L1, and PD-L2 signaling (Fig. S5F and G). Specifically, these TAMs exhibited selective enrichment of *TNFSF13B* (BAFF), targeting *TNFRSF13C* (BAFF-R) on malignant B cells (Fig. 4E and F). To verify the specificity of this axis, we performed rigorous correlation analyses. While bulk datasets confirmed a positive correlation, single-cell analysis revealed a stronger correlation between *TNFRSF1A* and *TNFSF13B* within macrophages (Fig. S6A to C). In contrast, *TNFRSF1A* expression in DCs showed no correlation with macrophage-derived *TNFSF13B*, suggesting that this axis is driven by macrophage-intrinsic regulation (Fig. S6D). Concurrently, TNFRSF1A^+^ TAMs showed elevated expression of *CD274* (PD-L1) and *PDCD1LG2* (PD-L2), interacting with *PDCD1* (PD-1) on CD8^+^ T cells and regulatory T cells, thereby establishing a dual mechanism of tumor support and immune evasion (Fig. 4G to J). To further validate these findings at the pathway level, we performed GSEA. Bulk GSEA demonstrated significant enrichment of TNF signaling via NF-κB, PD-L1 expression and PD-1 checkpoint pathway in cancer, and cytokine–cytokine receptor interaction, antigen processing and presentation in *TNFRSF1A*-high tumors (Fig. S5B). Consistently, single-cell GSEA comparing TNFRSF1A^+^ and TNFRSF1A^−^ TAMs confirmed enhanced activation of NF-κB signaling and PD-L1/PD-1 checkpoint pathways in the TNFRSF1A^+^ subset, defining a protumorigenic and immunomodulatory state (Fig. 4B).

To validate the spatial relevance of TNFRSF1A^+^ TAMs, we first defined a specific transcriptomic signature comprising 48 marker genes (*avg_log*_*2*_*FC* > 0.25, *p.adj* < 0.05) derived from our single-cell atlas (Table S3). We then analyzed a digital spatial profiling dataset (GSE232853) containing CD68^+^ macrophage segments. Consistent with our single-cell findings, *TNFRSF1A* expression was significantly elevated in DLBCL tissues compared to rLNs (Fig. 4M and Fig. S6E). Spatially, this TNFRSF1A^+^ TAM signature showed positive correlations with *TNFSF13B* (BAFF) and *CD274* (PD-L1) expression within tumor regions (Fig. 4N and Fig. S6F to H). We further mapped this signature against 6 previously defined spatial macrophage phenotypes (MacroSigs). The TNFRSF1A^+^ TAM signature exhibited the strongest correlation with MacroSig6 (*R* = 0.85), a DLBCL-specific subset associated with poor prognosis, followed by the interfollicular MacroSig2 (Fig. 4O). Pathway analysis indicated that both the TNFRSF1A-high (MacroSig6) and BAFF-high (MacroSig2) niches were enriched for TNF-α/NF-κB signaling. These data suggest that TNFRSF1A^+^ TAMs reside in specific tumor niches and maintain an inflammatory profile linked to immune suppression and tumor support.

**Fig. 5.**
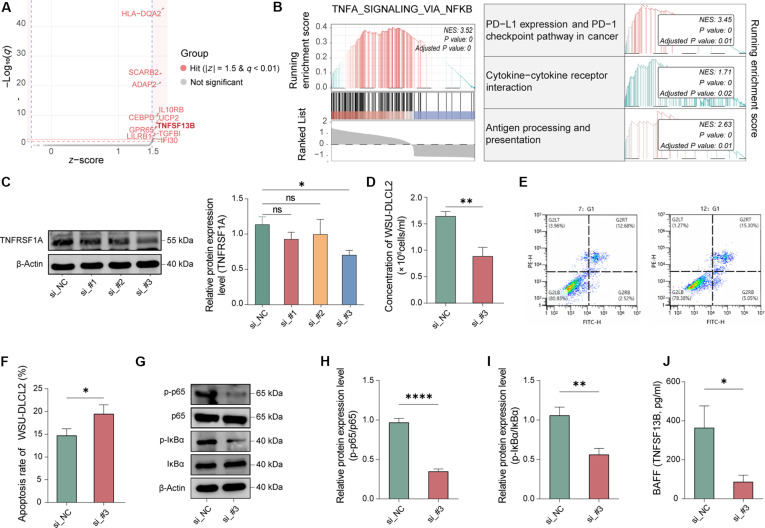
Computational and genetic perturbation validates tumor necrosis factor receptor superfamily member 1A (*TNFRSF1A*) as a key regulator of the tumor-associated macrophage (TAM)-mediated prosurvival axis. (A and B) In silico perturbation via scTenifoldKnk virtual knockout of *TNFRSF1A*: (A) volcano plot of predicted differentially expressed genes (DEGs) and (B) gene set enrichment analysis (GSEA) of WT versus pseudo-knockout (KO) networks. (C) Experimental validation of TNFRSF1A knockdown efficiency in phorbol 12-myristate 13-acetate (PMA)-differentiated THP-1 macrophages (M-THP1) by Western blot. (D) Proliferation of WSU-DLCL2 cells in Transwell coculture with control (si-NC) or *TNFRSF1A*-depleted (si-*TNFRSF1A*) macrophages. (E and F) Representative flow cytometry plots (E) and quantification (F) of apoptosis in WSU-DLCL2 cells after coculture. (G to I) Representative Western blot bands (G) and quantification of p-p65/p65 (H) and p-IκBα/IκBα (I) phosphorylation ratios in M-THP1 cells. (J) Concentration of secreted B-cell activating factor (BAFF) in coculture supernatants measured by enzyme-linked immunosorbent assay (ELISA). *P < 0.05, **P < 0.01, ****P < 0.0001; ns, not significant.

To transition from computational inference to provide functional validation, we first conducted a virtual knockout of *TNFRSF1A* in TAMs. This in silico perturbation resulted in a marked disruption of *TNFSF13B* expression (*z-score* = 1.62), along with reduced NF-κB and PD-L1/PD-1 signaling activities (Fig. 5A and B). Subsequently, we performed genetic perturbation in M-THP1 cells. Among 3 candidates, siRNA #3 exhibited the highest knockdown efficiency and was selected for downstream functional assays (Fig. 5C). In Transwell coculture experiments, targeted knockdown of *TNFRSF1A* in macrophages significantly impaired the survival advantage conferred to WSU-DLCL2 cells, leading to attenuated proliferation and increased apoptosis (Fig. 5D to F). Mechanistically, the phosphorylation of p65 and IκBα within macrophages was markedly inhibited following *TNFRSF1A* silencing, indicating the canonical NF-κB pathway was suppressed (Fig. 5G to I). Furthermore, ELISA confirmed that the secretion of BAFF into the coculture supernatant was significantly reduced in the si-*TNFRSF1A* group (Fig. 5J). These results demonstrate that *TNFRSF1A* directly regulates the NF-κB/BAFF axis to drive the TAM-mediated prosurvival microenvironment.

### Computational screening identifies curcumin as a potential modulator of TNFRSF1A

**Fig. 6.**
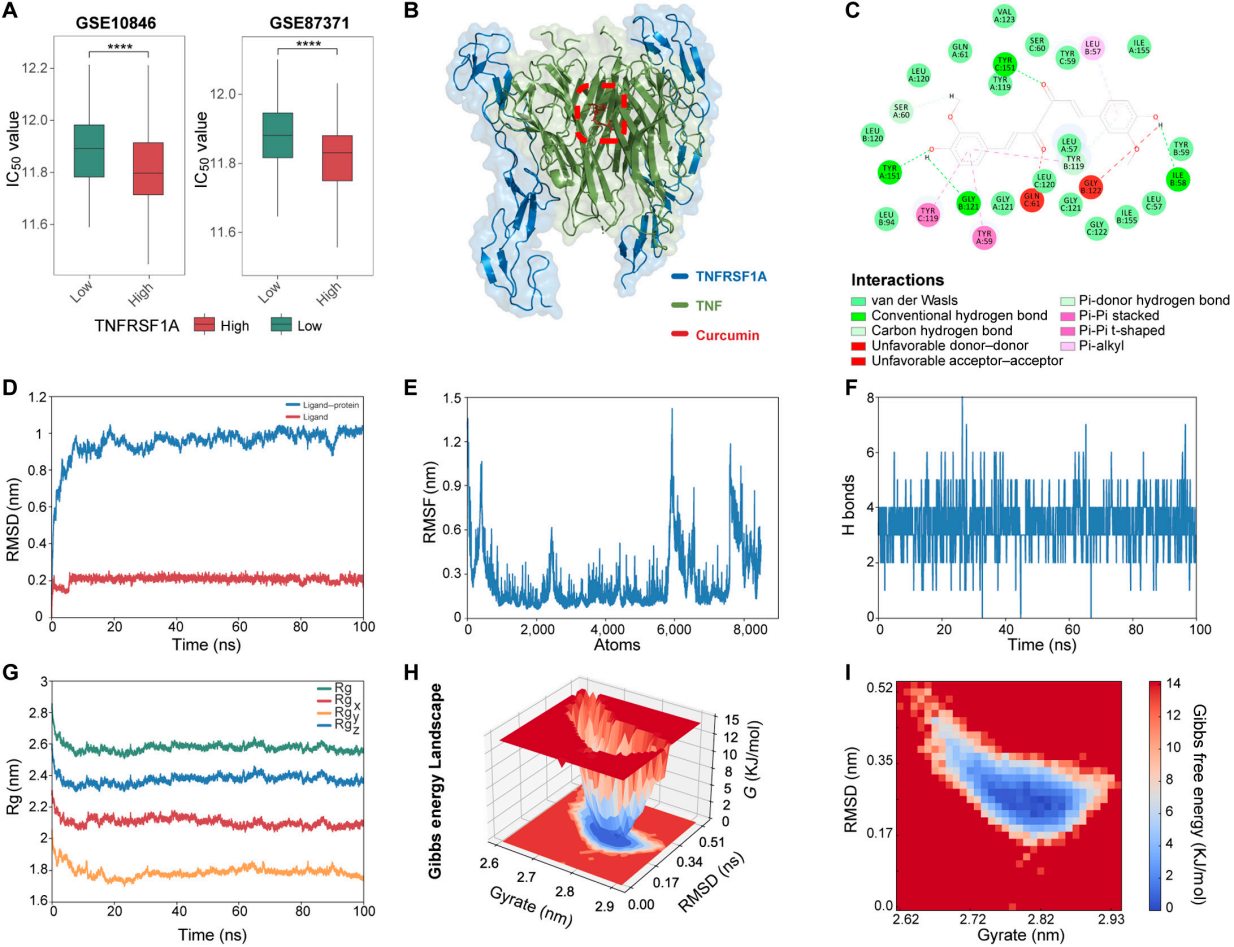
Identification and structural validation of curcumin as a potential tumor necrosis factor receptor superfamily member 1A (TNFRSF1A) modulator. (A) Pharmacogenomic prediction of curcumin half-maximal inhibitory concentration (*IC*_*50*_) values in TNFRSF1A-high versus -low groups across validation cohorts. (B and C) Binding characterization: (B) predicted 3-dimensional (3D) binding mode of curcumin at the tumor necrosis factor (TNF)–TNFRSF1A interface and (C) 2D diagram of protein-ligand interactions. (D to G) 100 ns molecular dynamics (MD) simulation analysis: (D) Root-mean-square deviation (RMSD) profiles of the complex and curcumin, (E) protein root-mean-square fluctuation (RMSF), (F) temporal hydrogen bond occupancy, and (G) radius of gyration (Rg) reflecting structural compactness. (H and I) Thermodynamic stability assessment: (H) 3D and (I) 2D projection of the Gibbs free energy landscape. ****P < 0.0001.

Given the key role of *TNFRSF1A* in orchestrating the protumorigenic niche, we sought to identify small-molecule modulators targeting this receptor. In our preceding pharmacogenomic screening, we observed that high *TNFRSF1A* expression was associated with significantly increased sensitivity (lower IC_50_) to curcumin across 2 independent DLBCL cohorts (GSE10846 and GSE87371) (Fig. 6A). This significant association suggests that TNFRSF1A-overexpressing tumors are susceptible to curcumin, identifying curcumin as a potential candidate for therapeutic intervention. The crystal structure of the TNF–TNFRSF1A complex (PDB ID: 7kpb) reveals that the asymmetric human TNF trimer binds 2 TNFRSF1A molecules instead of 3 in the presence of a small-molecule inhibitor, thereby impairing its signaling function [35]. To investigate the binding mechanism and stability of curcumin as a small-molecule inhibitor in the TNF–TNFRSF1A signaling, we evaluated its binding energy based on the complex crystal structure. In the characterized inhibitory pocket, curcumin exhibited strong binding affinity, although weaker than the reference inhibitor (−8.68 kcal/mol *vs.* −10.2 kcal/mol), stabilized through hydrogen bonds with conserved residues Tyr151, Gly121, and Ile58 (Fig. 6B and C).

MD simulations were employed to characterize the binding interactions and dynamic stability of small molecule–protein complexes [36]. To probe dynamic behavior, we performed a 100-ns MD simulation. Key findings include protein backbone RMSD stabilizing at 0.9 to 1.1 nm post-10-ns equilibration and ligand RMSD converging to 0.2 nm, indicating kinetic binding stability (Fig. 6D). RMSF analysis revealed 3 flexible regions (atoms ~400, 2,400, and 5,900) at intersubunit junctions, while curcumin-binding residues displayed low fluctuations, further confirming stability (Fig. 6E). Hydrogen bond occupancy averaged 4 ± 2 hydrogen bonds (peak at 8 bonds), supporting moderate-affinity binding (Fig. 6F). The radius of gyration remained stable at 2.6 ± 0.1 nm, indicating stable structural compactness of the complex (Fig. 6G). The Gibbs free energy landscape analysis identified a dominant low-energy basin, affirming the thermodynamic stability of the curcumin-bound state (Fig. 6H and I). These results suggest that given its stable binding mode, curcumin may interact with TNF signaling via TNFRSF1A.

### Curcumin suppresses DLBCL progression by disrupting the TAM-mediated TNFRSF1A/NF-κB/BAFF axis

Following the genetic validation of the TNFRSF1A/BAFF axis and the computational prediction of curcumin as a potential modulator, we next investigated whether curcumin could functionally disrupt this macrophage–tumor crosstalk. We first determined the IC_50_ in phorbol 12-myristate 13-acetate-differentiated THP-1 macrophages (M-THP1) and WSU-DLCL2 cells, selecting a subcytotoxic dose of 15 μM for downstream coculture investigations (Fig. S7A and B). While coculture induced a significant up-regulation of TNFRSF1A protein in macrophages, curcumin treatment failed to significantly reduce TNFRSF1A protein levels (Fig. S7C). This observation is consistent with our molecular docking results, suggesting that curcumin may act as a potential modulator of *TNFRSF1A* signaling without directly affecting its protein stability.

**Fig. 7.**
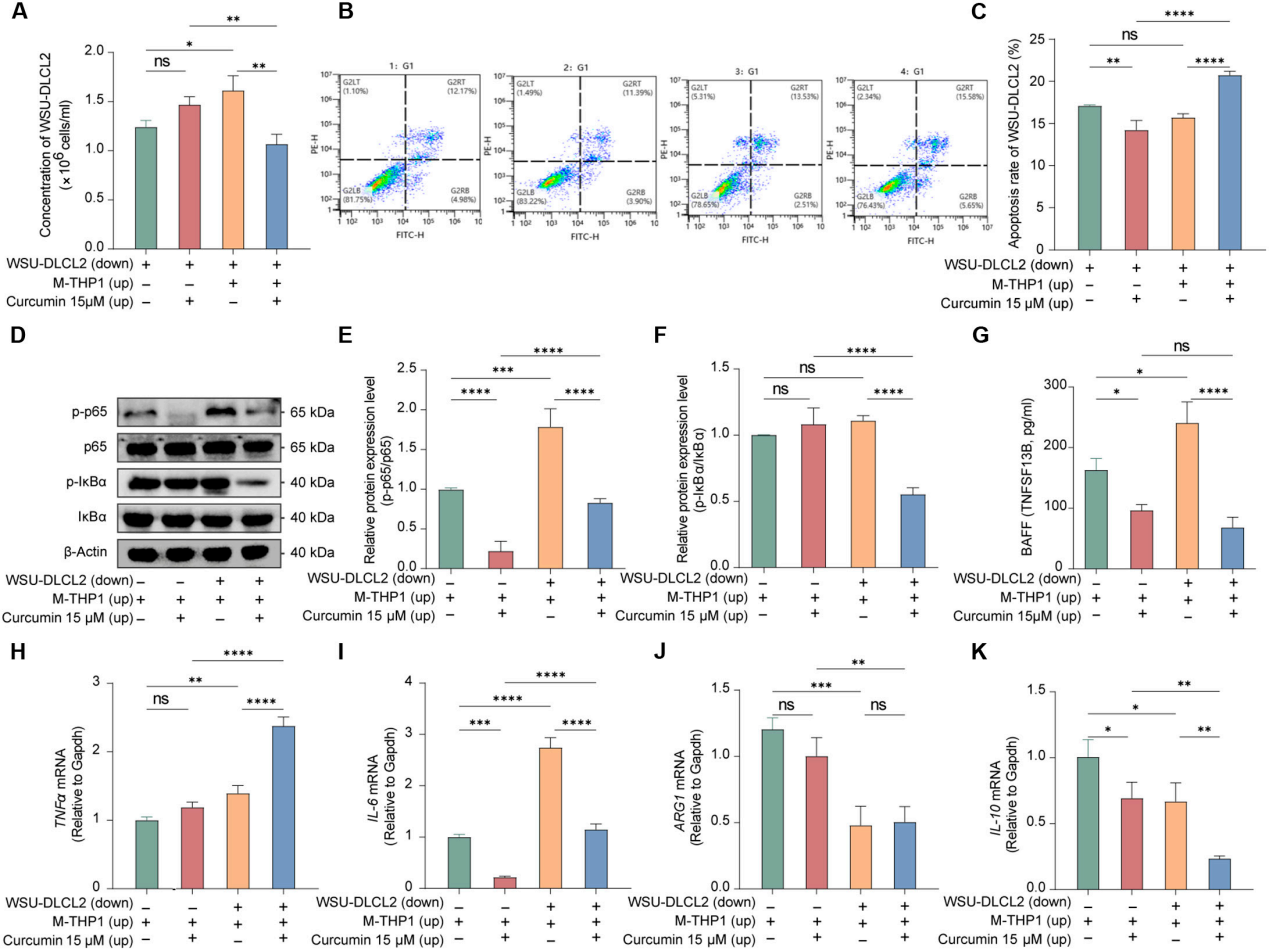
Functional validation confirms curcumin reverses tumor-associated macrophage (TAM)-mediated tumor cell activation in coculture systems. (A) Bar graph showing tumor cell concentration across different treatment groups. (B) Representative flow cytometry plots of cell apoptosis in different groups. (C) Bar graph showing the apoptosis rate (%) in different groups. (D) Representative Western blot bands of total and phosphorylated nuclear factor-κB (NF-κB) components in different groups. (E) Bar graph quantifying the relative levels of p-p65 normalized to total p65 across groups. (F) Bar graph quantifying the p-IκBα/IκBα phosphorylation ratio across different groups. (G) Bar graph illustrating B-cell activating factor (BAFF) concentration in coculture supernatants measured by enzyme-linked immunosorbent assay (ELISA). (H to K) Relative mRNA levels of tumor necrosis factor α (TNFα) (H), interleukin-6 (IL-6) (I), arginase 1 (ARG1) (J), and IL-10 (K) were measured in different groups using reverse transcription quantitative polymerase chain reaction (RT-qPCR). *n* = 3, *P < 0.05, **P < 0.01, ***P < 0.001, ****P < 0.0001; ns, not significant.

We utilized a Transwell coculture system to assess the effect of curcumin on the interaction between TAMs and lymphoma cells. Coculture with M-THP1 macrophages provided a significant survival advantage to WSU-DLCL2 cells, characterized by enhanced proliferation and attenuated apoptosis, while these protective effects were reversed by curcumin treatment (Fig. 7A to C). Mechanistically, Western blotting revealed that the coculture microenvironment triggered strong activation of the NF-κB pathway in macrophages, with increased phosphorylation of p-p65 and p-IκBα, which coincided with a surge in BAFF secretion. Curcumin treatment potently suppressed NF-κB phosphorylation and reduced BAFF secretion, thereby dismantling the prosurvival signaling loop (Fig. 7D to G). Furthermore, reverse transcription-qPCR analysis demonstrated that curcumin modulated the cytokine profile of TAMs, selectively suppressing the protumorigenic cytokine IL-6 and the immunosuppressive marker IL-10, without significantly affecting *TNFα* and *ARG1* expression (Fig. 7H to K). Notably, the inhibitory effects of curcumin on the macrophage-mediated NF-κB/BAFF axis closely recapitulated the phenotype observed following genetic silencing of *TNFRSF1A*, suggesting that this receptor is a key functional mediator of curcumin’s impact on the tumor-supportive microenvironment.

### Construction of a TNFRSF1A^+^ TAM-related prognostic model

**Fig. 8.**
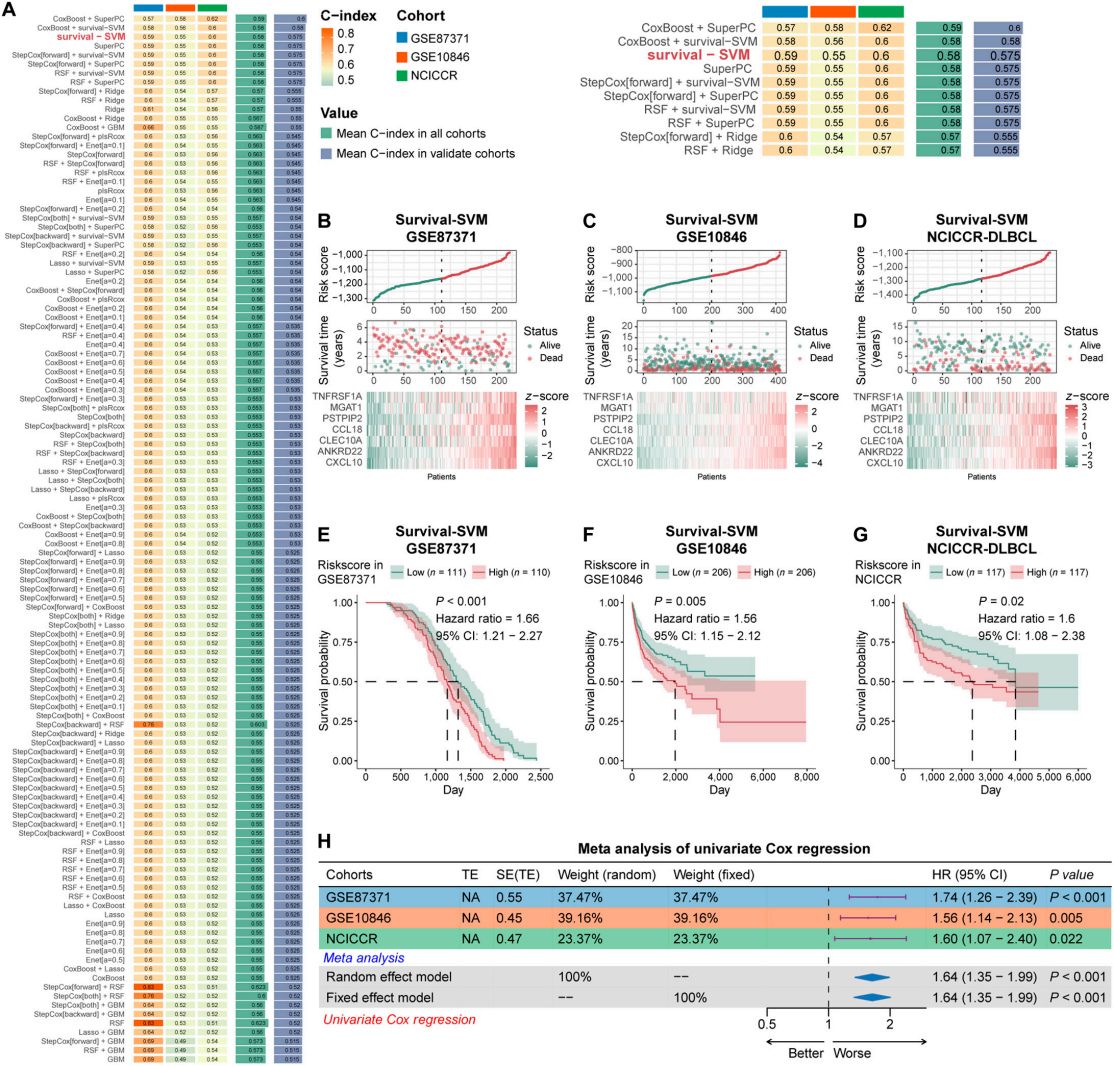
Construction and validation of a tumor necrosis factor receptor superfamily member 1A-positive (TNFRSF1A^+^) tumor-associated macrophage (TAM)-derived prognostic signature in diffuse large B-cell lymphoma (DLBCL). (A) Benchmark analysis of 101 machine learning algorithm combinations in GSE87371 and GSE10846 cohorts, highlighting “StepCox[forward] + RSF” as best-performing model. (B and C) Risk score distribution, survival status, and expression profiles of the 12 signature genes in GSE87371 (B) and GSE10846 (C). (D and E) Kaplan–Meier curves for overall survival in high-risk and low-risk groups in GSE87371 (D) and GSE10846 (E). (F) Forest plot of univariate Cox regression and meta-analysis evaluating the prognostic value of the risk score across the 2 datasets.

To translate our findings into clinical utility, we utilized the TNFRSF1A^+^ TAM gene signature to construct a prognostic model. Using the Mime framework, we benchmarked 101 combinations of machine learning algorithms across 3 independent cohorts (GSE87371, GSE10846, and NCICCR-DLBCL). Based on the concordance index (C-index), Survival-SVM was identified as the optimal modeling strategy (Fig. 8A). This approach identified a 7-gene signature, including *TNFRSF1A*, *CXCL10*, *ANKRD22*, *CLEC10A*, *CCL18*, *PSTPIP2*, and *MGAT1* (Table S4). High-risk scores were associated with increased mortality and distinct gene expression profiles (Fig. 8B to D). Kaplan–Meier analyses confirmed that patients in the high-risk group exhibited significantly inferior overall survival across all 3 cohorts (GSE87371: *HR* = 1.35, *P* = 0.046; GSE10846: *HR* = 1.52, *P* = 0.008; NCICCR-DLBCL: *HR* = 1.54, *P* = 0.033) (Fig. 8E to G). Moreover, univariate Cox regression and meta-analysis further identified the risk score as a significant predictor (*HR* = 1.64, *P* < 0.001) (Fig. 8H). Finally, time-dependent receiver operating characteristic curves, calibration plots, and decision curve analysis validated the model’s predictive accuracy, calibration, and clinical net benefit for 1-, 3-, and 5-year survival (Figs. S8 and S9). In summary, this signature provides a reliable tool for risk stratification and confirms the role of TNFRSF1A^+^ TAM in DLBCL progression.

## Discussion

**Fig. 9.**
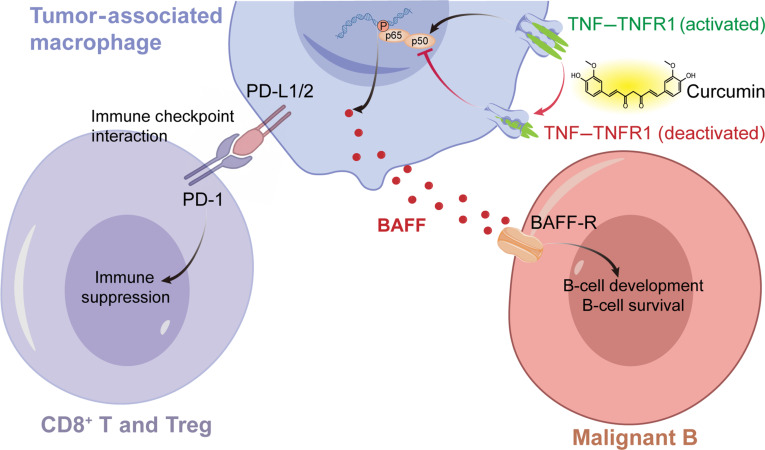
Schematic illustration of tumor necrosis factor receptor superfamily member 1A-positive (TNFRSF1A^+^) tumor-associated macrophage (TAM)-mediated signaling in diffuse large B-cell lymphoma (DLBCL).

While DLBCL progression is closely linked to immunosuppression mediated by TAMs, the molecular drivers regulating TAM functional states remain largely undefined [37]. In this study, we employed an integrative multiomics approach alongside experimental validation to characterize TNFRSF1A^+^ TAMs as modulators of therapeutic resistance in DLBCL (Fig. 9). Unlike previous studies centered on tumor-intrinsic B-cell receptor/NF-κB signaling in ABC-DLBCL, we demonstrate that TNFRSF1A acts as a regulator within TAMs [38]. It maintains a supportive microenvironment by driving NF-κB-dependent BAFF secretion to promote malignant B-cell fitness and PD-L1 expression to facilitate T-cell exhaustion. Furthermore, curcumin potentially modulates this macrophage-intrinsic TNFRSF1A/NF-κB/BAFF axis to attenuate macrophage-mediated support for lymphoma cell survival.

Our findings expand the current understanding of TAM heterogeneity in the lymphoma microenvironment. While seminal studies have firmly established that TAM infiltration, particularly those exhibiting M2-like features, correlates with poor prognosis in R-CHOP-treated patients, the functional plasticity of these cells likely transcends the classical binary M1/M2 paradigm [39]. Consistent with recent single-cell transcriptomic analyses that have revealed the inherent complexity of TAMs, including interferon-primed or C1q-expressing subsets, our work refines this landscape by demonstrating that TNFRSF1A-high TAMs represent a critical functional state rather than an entirely new macrophage lineage [40,41]. The novelty of our findings lies in integrating TNFRSF1A with the NF-κB–BAFF/PD-L1 signaling axis and its association with therapeutic resistance. Unlike the classical view where TNFRSF1A signaling shifts between cell survival and apoptosis depending on Complex I/II assembly, we show that in DLBCL TAMs, TNFRSF1A signaling is predominantly skewed toward a prosurvival, NF-κB-hyperactivated state [12]. While parallel pathways such as Toll-like receptors also contribute to macrophage activation, our data highlight TNFRSF1A as a important and druggable node within this complex regulatory network. This aligns with observations that the chronic inflammatory milieu in lymphomas favors persistent NF-κB activation over cell death [42]. Importantly, the link between TNFRSF1A and predicted vincristine resistance explains why pan-macrophage depletion fails to yield clinical benefits.

Our mechanistic investigations revealed a paracrine survival loop wherein TNFRSF1A^+^ TAMs secrete BAFF to support malignant B cells, while simultaneously expressing PD-L1/PD-L2, which may attenuate T-cell immunity. The established role of BAFF as a survival factor is well documented, and elevated serum BAFF levels have been directly linked to R-CHOP resistance and increased relapse rates in DLBCL patients [43]. Our data refine this model by identifying a key upstream regulator: It is the TNFRSF1A-mediated activation of the canonical NF-κB pathway that drives *TNFSF13B* (BAFF) transcription. This finding aligns with established literature in B-cell-mediated pathologies, such as rheumatoid arthritis, where the BAFF signaling axis is mechanistically associated with NF-κB pathway activation to drive B-cell proliferation and survival [44]. Furthermore, distinct from studies attributing TAM-mediated survival support primarily to direct cell-contact mechanisms like the CD47–SIRPα (signal-regulatory protein α) axis, our work emphasizes the importance of a parallel paracrine signaling axis (TNF–TNFRSF1A–NF-κB–BAFF) [45]. Finally, our observation that TNFRSF1A^+^ TAMs coexpress PD-L1 supports the concept that a single regulatory node can concurrently regulate tumor growth support and immune evasion, highlighting TNFRSF1A as a potential high-priority therapeutic target.

Therapeutically, our study repositions curcumin from a general anti-inflammatory agent to a potential antagonist of the TNFRSF1A signaling complex. While curcumin has long been recognized for its ability to inhibit NF-κB pathways in tumor cells and to reprogram M2 macrophages toward an M1 phenotype, the exact molecular targets are not fully characterized [46,47]. Our MD simulations provide structural evidence suggesting that curcumin may occupy a functional pocket within the TNFRSF1A receptor complex. This structural insight is consistent with the marked downstream effects observed in our coculture experiments, where curcumin treatment recapitulated the effects of TNFRSF1A silencing—the down-regulation of NF-κB signaling and the subsequent reduction in BAFF secretion. By demonstrating that curcumin can disrupt the macrophage-mediated supportive niche, our work provides a mechanistic rationale for its use as a potential adjuvant to R-CHOP, particularly in patients with high TNFRSF1A^+^ TAM infiltration who are at predicted risk of vincristine resistance.

While this study provides novel insights into the DLBCL microenvironment, several limitations should be acknowledged. First, although siRNA-mediated perturbation provides causal evidence *in vitro*, our Transwell system—relying on established cell lines—primarily captures paracrine interactions and lacks the 3-dimensional architecture and *in vivo* complexity of the TME. Future studies utilizing macrophage-specific knockout models, primary patient-derived samples, and 3-dimensional organoids are needed to fully validate this axis. Second, because the digital spatial profiling dataset lacks true single-cell spatial coordinates, our spatial analyses are limited to niche-level correlations. Future investigations utilizing high-resolution spatial molecular imaging will be necessary to quantify the direct physical proximity between TNFRSF1A^+^ TAMs and malignant cells. Third, because we did not perform direct biophysical binding assays (e.g., surface plasmon resonance or isothermal titration calorimetry), curcumin’s interaction with TNFRSF1A remains computationally inferred. Given curcumin’s pleiotropic nature and our reliance on a single-structure MD simulation, we cannot exclude synergistic effects through other pathways (e.g., IκB kinase or signal transducer and activator of transcription 3) or receptor conformational plasticity. Subsequent research with more selective antagonists like R-7050 will be essential to isolate receptor-specific contributions. Finally, while our prognostic signature is robust across multiple retrospective cohorts and mitigates the risk of overfitting, our chemoresistance findings rely on algorithmic predictions rather than explicit clinical response data. Prospective clinical trials remain necessary to confirm its utility in guiding therapeutic decisions. Ultimately, these strategies could facilitate the development of therapies targeting the macrophage-supported niche that promotes DLBCL progression and chemoresistance.

In conclusion, this study identifies TNFRSF1A as a functional marker of a specific TAM subset in DLBCL and establishes its role in regulating microenvironmental interactions that promote lymphoma persistence. We characterized a signaling axis wherein TNFRSF1A activates the NF-κB pathway to drive a paracrine BAFF/PD-L1 network, thereby mediating both tumor survival and immune evasion. Importantly, our pharmacogenomic and experimental evaluation identifies curcumin as a potential therapeutic agent capable of disrupting this macrophage-dependent support system. By translating these findings into a prognostic signature, we provide a clinical tool for stratifying high-risk patients who may benefit from TAM-targeted interventions. These insights not only expand our knowledge of the lymphoma microenvironment but also suggest a potential strategy to overcome chemoresistance in the clinical setting.

## Data Availability

The datasets supporting the conclusions of this article are available in public repositories as follows. Bulk transcriptomic data: The GSE56315, GSE25638, GSE10846, and GSE87371 datasets are available in the Gene Expression Omnibus (GEO) repository (https://www.ncbi.nlm.nih.gov/geo/). The TCGA-DLBC dataset was obtained from the Genomic Data Commons Data Portal (https://portal.gdc.cancer.gov/). Single-cell RNA sequencing data: The GSE182434 dataset is available in the GEO repository. The heiDATA-VRJUNV dataset was obtained from the heiDATA repository (https://heidata.uni-heidelberg.de/dataset.xhtml?persistentId=doi:10.11588/data/VRJUNV). Spatial transcriptomics data: The macrophage-related spatial signatures (MacroSigs) were derived from the GSE232853 dataset available in the GEO repository. All data generated or analyzed during this study are included in this published article and its supplementary information files.
