# Supplementary material for: Integrative Multiomics and Single-Cell Profiling Identify TNFRSF1A+ Macrophages as a Prognostic and Therapeutic Target in Diffuse Large B-cell Lymphoma
Source: Comput Struct Biotechnol J. 2026 Jun 1;35(1):0119. doi: 10.34133/csbj.0119 (PMC13223359; doi:10.34133/csbj.0119)
Supplement: Supplementary 1 — Figs. S1 to S9 Tables S1 to S4 [file csbj.0119.f1.docx]

Title

Integrative Multi-omics and Single-cell Profiling Identify TNFRSF1A^+^ Macrophages as a Prognostic and Therapeutic Target in Diffuse Large B-cell Lymphoma

**Authors**

Wenxian Yin ^1,2,3†^, Xingyue Wang ^3†^, Zhuo Chen ^4^, Mengqi Sun ^5^, Yinping Zhang ^3^, Jiahong Su ^3^, Zijun Yuan ^3^, Ting Xiao ^1^, Singkome Tima ^1^, Xi He ^4^, Zhangang Xiao ^3,6,7*^, Suwit Duangmano ^1*^

**Affiliations**

^1^ Department of Medical Technology, Faculty of Associated Medical Sciences, Chiang Mai University, Chiang Mai 50200, Thailand.

^2^ Department of Pharmacy, The Affiliated Traditional Chinese Medicine Hospital, Southwest Medical University, Luzhou 646000, China

^3^ Laboratory of Molecular Pharmacology, Department of Pharmacology, School of Pharmacy, Southwest Medical University, Luzhou 646000, China

^4^ Infectious Disease Department, The Six School of Clinical Medicine, the Affiliated Qingyuan Hospital (Qingyuan People’s Hospital), Guangzhou Medical University, Guangdong 511518, China

^5^ Drug Clinical Trial Institution, the Affiliated Hospital, Southwest Medical University, Luzhou 646000, China

^6^ Cell Therapy & Cell Drugs of Luzhou Key Laboratory, Luzhou 646000, China

^7^ South Sichuan Institute of Translational Medicine, Luzhou 646000, China

^*^ Address correspondence to: zhangangxiao@swmu.edu.cn (Zhangang Xiao), suwit.du@cmu.ac.th (Suwit Duangmano)

^†^ These authors contributed equally to this work.


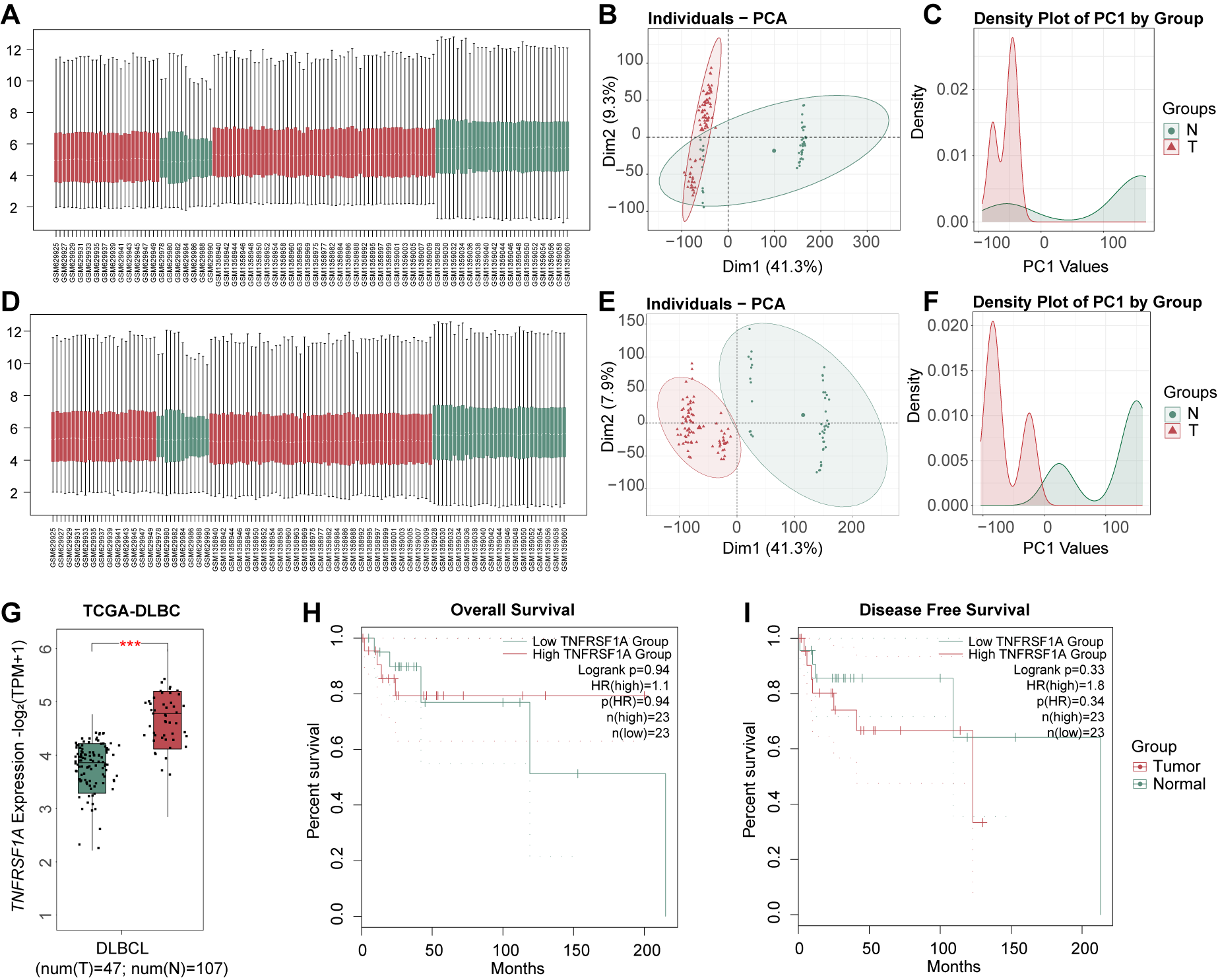


***Fig. S1.*** *Validation of TNFRSF1A prognostic significance and data preprocessing. (A–F) Evaluation of batch effect removal in discovery cohorts: (A–C) before and (D–F) after correction, visualized via gene expression distributions, PCA plots, and density plots. (G) Comparative analysis of TNFRSF1A mRNA expression between DLBCL and normal controls in the TCGA-DLBC cohort. (H–I) Kaplan-Meier curves for (H) overall survival and (I) disease-free survival according to TNFRSF1A expression in the TCGA-DLBC cohort. ^*^p < 0.05, ^**^p < 0.01, ^***^p < 0.001, ^****^p < 0.0001; ns, not significant.*


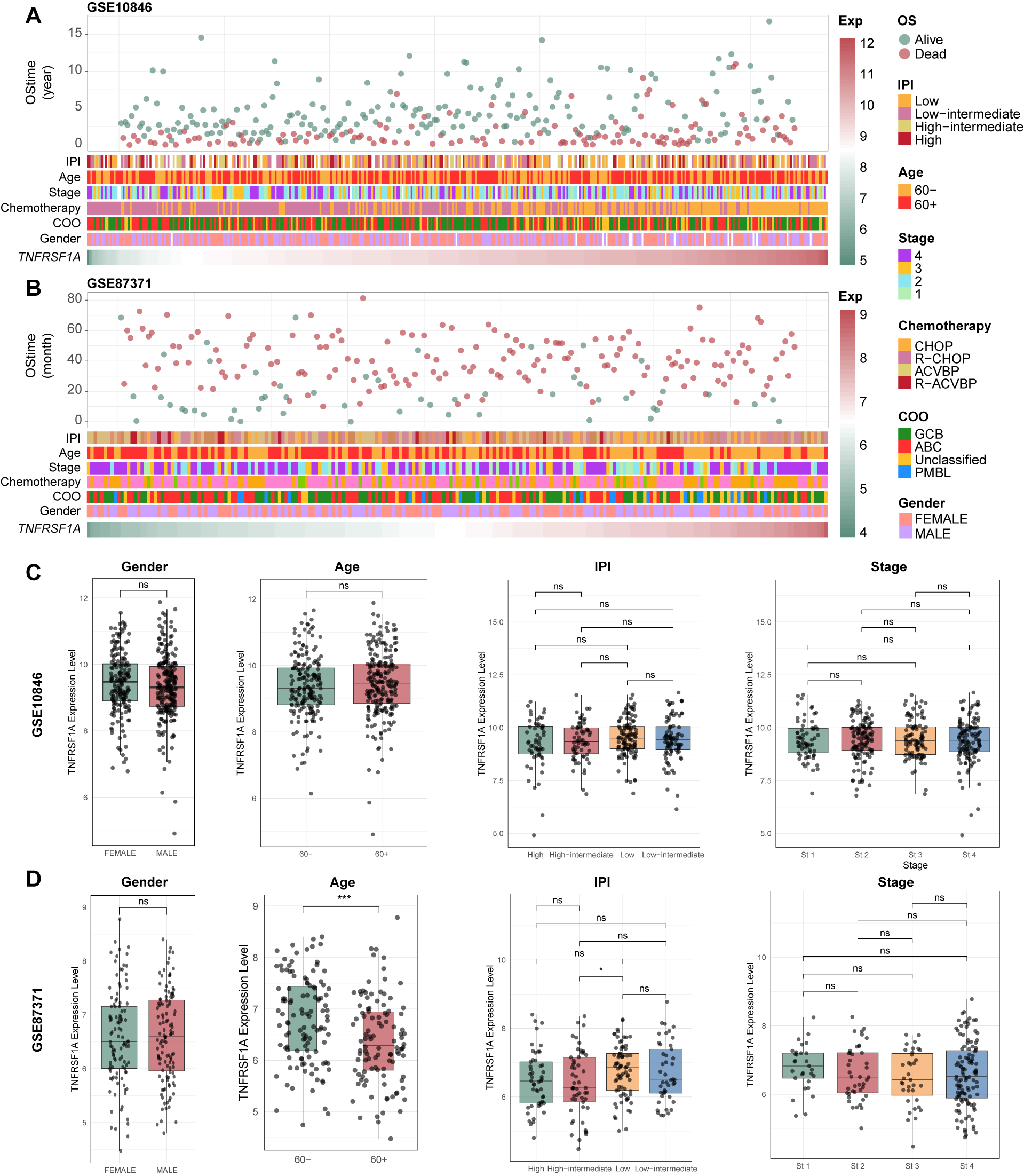


***Fig. S2.*** *Correlation of TNFRSF1A expression with clinicopathological characteristics in DLBCL. (A–B) Clinical landscape and TNFRSF1A expression profiles in validation cohorts (A) GSE10846 and (B) GSE87371. Scatter plots and heatmaps depict the distribution of survival status and clinicopathological features. (C–D) Association analysis of TNFRSF1A expression with key clinical parameters, including gender, age, IPI score, and Ann Arbor stage in (C) GSE10846 and (D) GSE87371. ^*^p < 0.05, ^**^p < 0.01, ^***^p < 0.001, ^****^p < 0.0001; ns, not significant.*


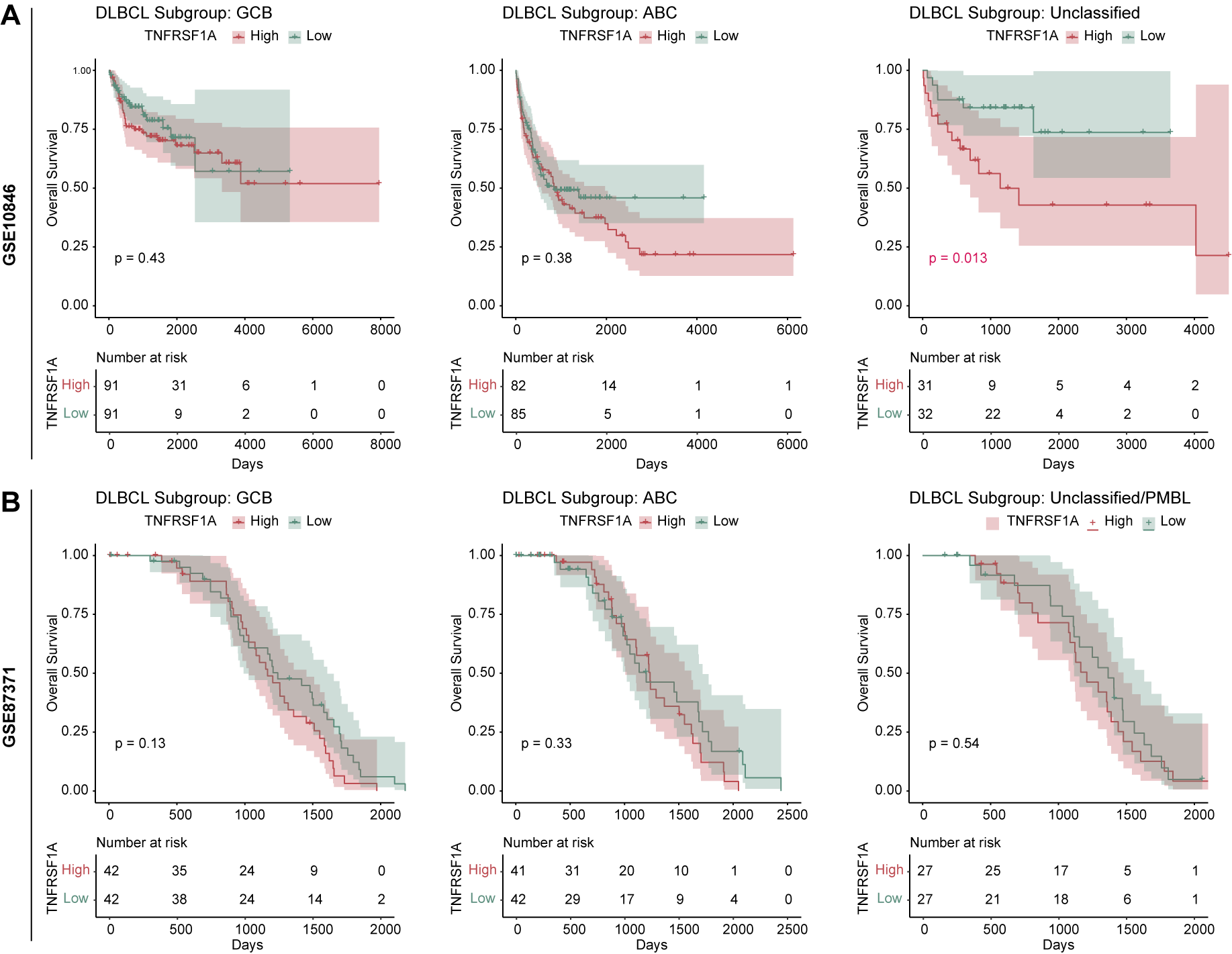


***Figure S3.*** *Prognostic impact of TNFRSF1A expression across COO subtypes in independent DLBCL cohorts. (A) Kaplan–Meier survival curves for overall survival (OS) stratified by TNFRSF1A expression (high vs. low) within germinal center B-cell-like (GCB), activated B-cell-like (ABC), and Unclassified subtypes in the GSE10846 cohort. (B) Kaplan–Meier survival curves for OS stratified by TNFRSF1A expression (high vs. low) within GCB, ABC, and Unclassified/primary mediastinal B-cell lymphoma (Unclassified /PMBL) subtypes in the GSE87371 cohort.*


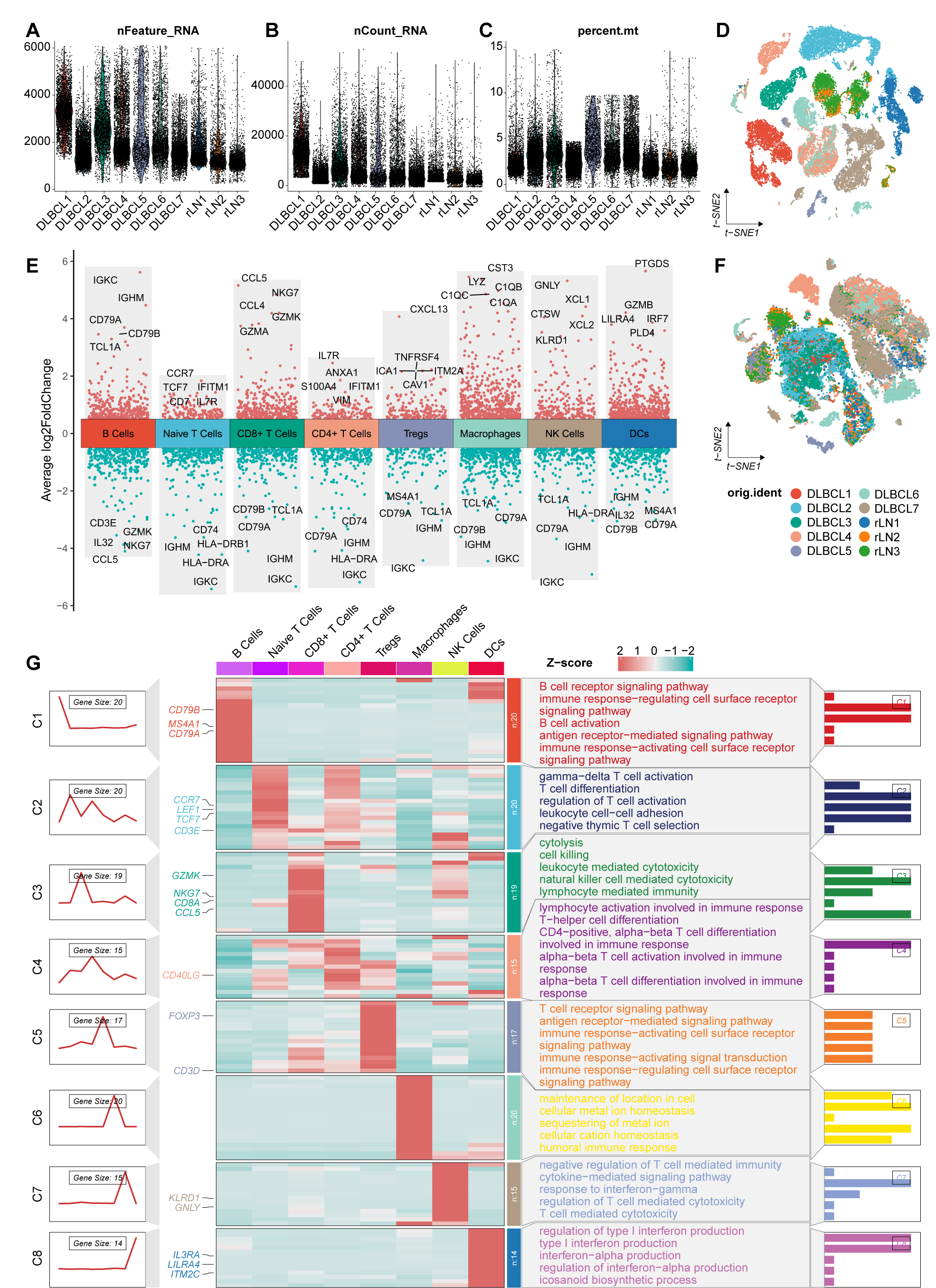


***Fig. S4.*** *Quality control and cell-type annotation validation of the scRNA-seq dataset. (A–C) Quality control metrics showing (A) number of gene features, (B) UMI counts, and (C) mitochondrial gene percentage across samples prior to filtering. (D, F) t-SNE visualization of cell distributions (D) before and (F) after CCA-based batch effect correction. (E) Volcano plots highlighting cluster-specific marker genes for the identified cell populations. (G) Heatmap of the top 20 marker genes per cluster and corresponding Gene Ontology (GO) biological process enrichment, validating the accuracy of cell-type identities.*


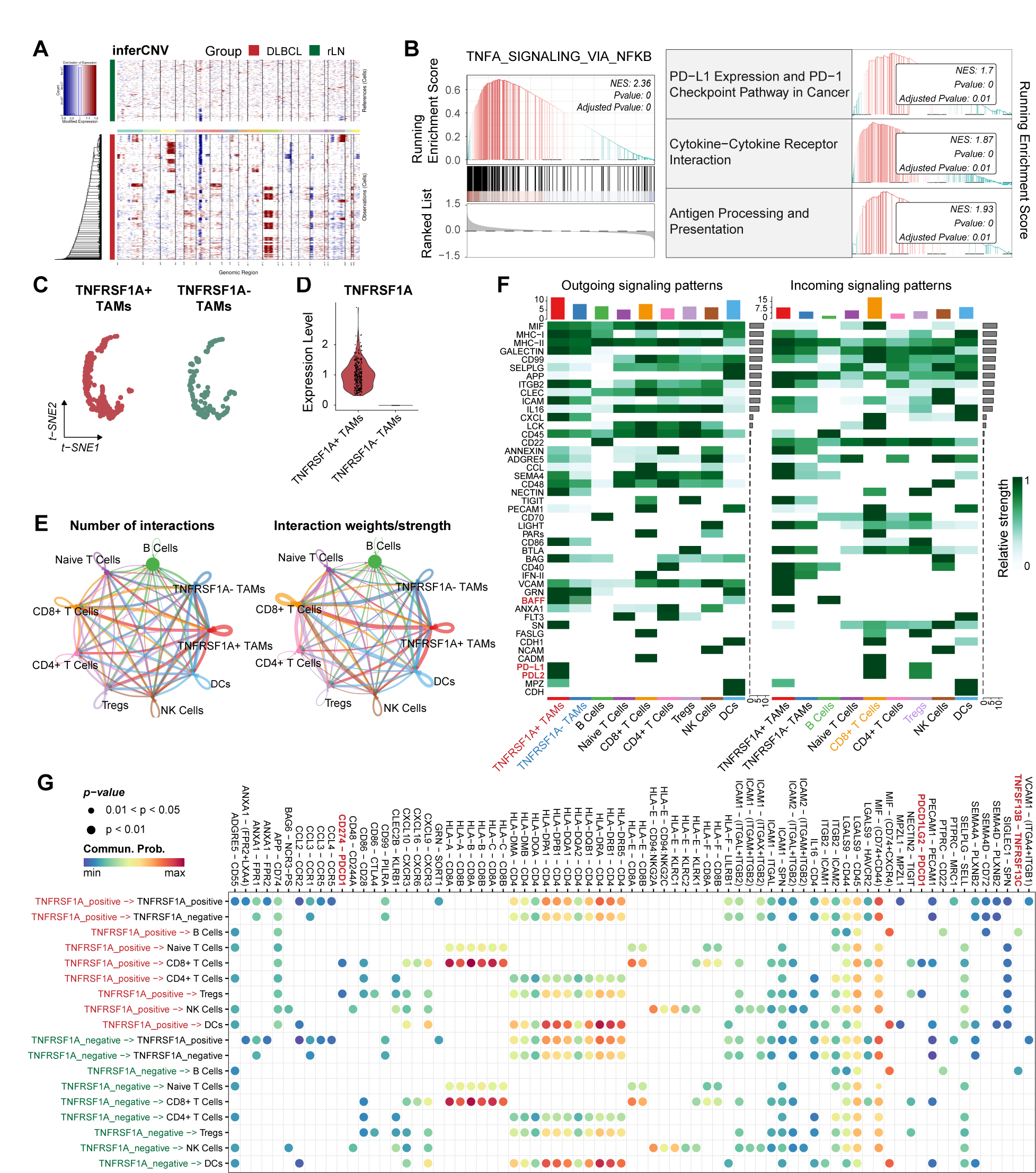


***Fig. S5.*** *Malignant cell identification and intercellular crosstalk analysis of TNFRSF1A^+^ TAMs. (A) InferCNV analysis characterizing large-scale chromosomal copy number variations in DLBCL B cells relative to rLN references. (B) GSEA of bulk transcriptomic data illustrating the significant enrichment of NF-κB, PD-L1, and immune-related pathways in TNFRSF1A-high patients. (C–D) Stratification of TAMs visualized by (C) t-SNE and (D) violin plots based on TNFRSF1A expression status. (E–G) Systematic intercellular communication modeling via CellChat: (E) global networks illustrating interaction frequency and weight among cell clusters, (F) heatmaps summarizing the relative contribution of outgoing and incoming signaling patterns, and (G) bubble plot highlighting specific ligand-receptor pairs involved in the crosstalk between TAM subsets and the DLBCL microenvironment.*


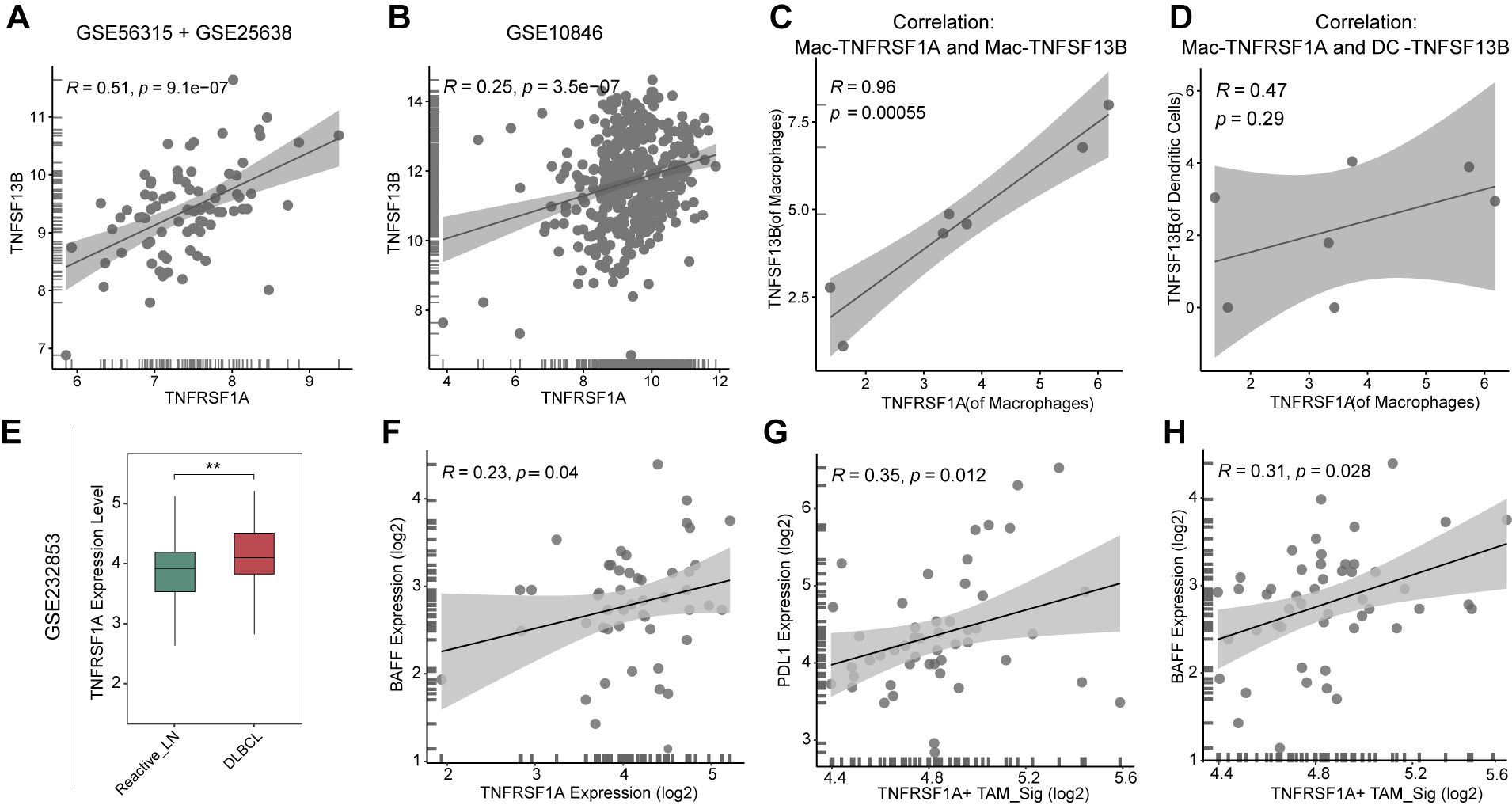


***Fig. S6.*** *Multi-omics validation of the TNFRSF1A/BAFF axis in the DLBCL microenvironment. (A–B) Spearman correlation between TNFRSF1A and TNFSF13B (BAFF) expression in bulk transcriptomic cohorts: (A) merged GSE56315 and GSE25638, and (B) GSE10846. (C–D) Specificity validation in scRNA-seq data highlighting (C) a robust association between TNFRSF1A and TNFSF13B within macrophages, contrasted with (D) a non-significant correlation in dendritic cells. (E–H) Spatial transcriptomic verification (GSE232853): (E) differential expression of TNFRSF1A in DLBCL versus reactive lymph nodes; (F) spatial correlation between TNFRSF1A and BAFF expression; (G–H) correlation of the scRNA-seq-derived TNFRSF1A^+^ TAM signature with (G) PD-L1 and (H) BAFF levels in the tumor niche. ^*^p < 0.05, ^**^p < 0.01, ^***^p < 0.001, ^****^p < 0.0001; ns, not significant.*


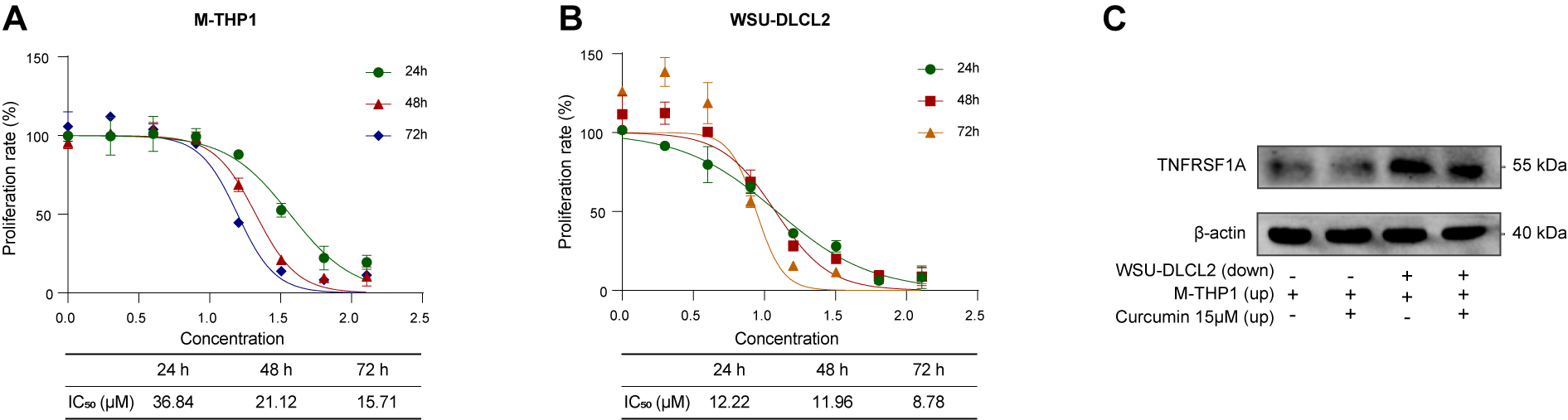


***Fig. S7.*** *Dose determination and effect of curcumin on TNFRSF1A protein expression. (A–B) CCK-8 cell viability assays determining the IC50 of curcumin in (A) M-THP1 macrophages and (B) WSU-DLCL2 cells after 24, 48, and 72 h of treatment. (C) Western blot analysis of TNFRSF1A protein expression in M-THP1 macrophages under different conditions.*


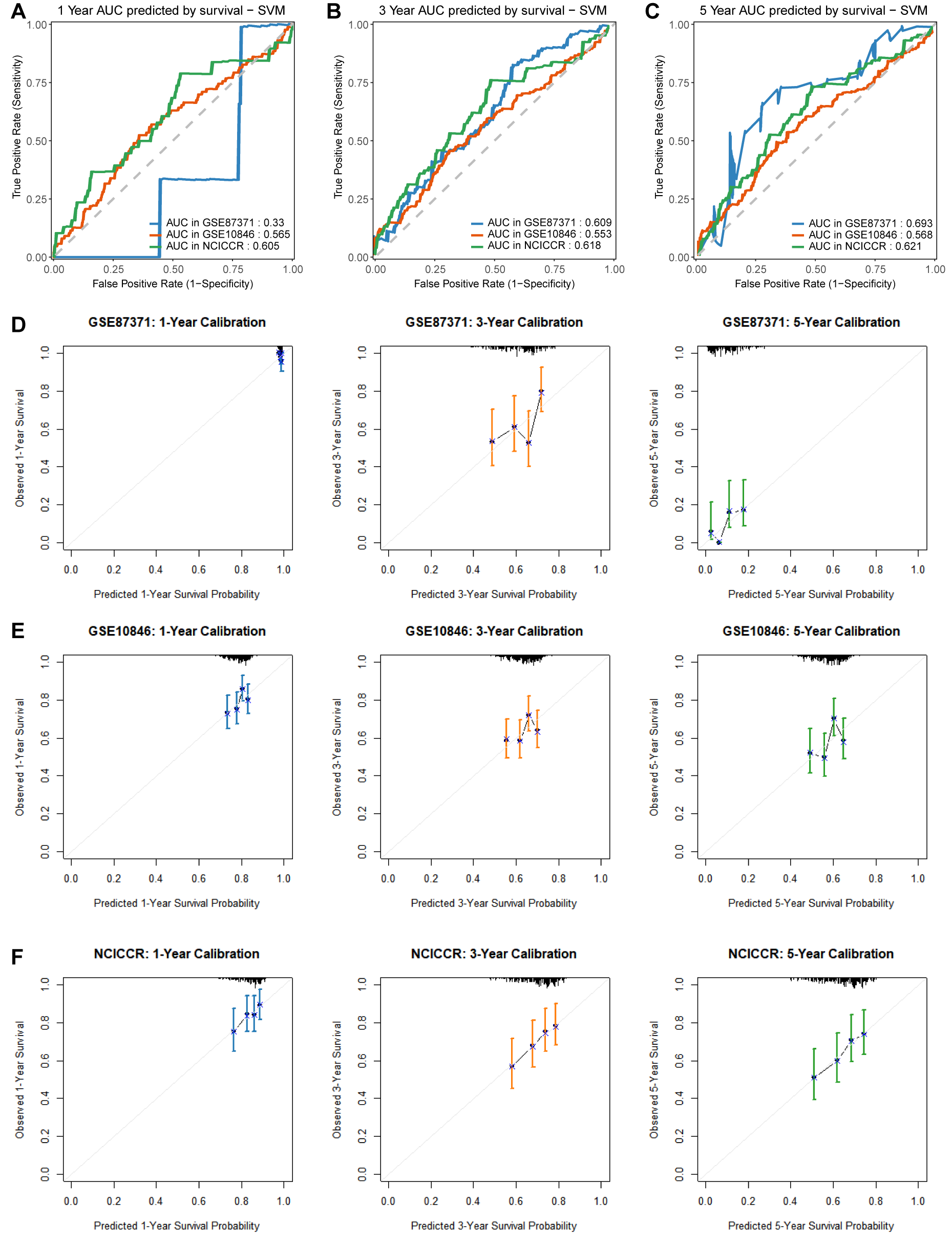


***Fig. S8.*** *Evaluation of the predictive performance and calibration of the prognostic signature. (A–C) Time-dependent ROC curves evaluating the predictive accuracy of the Survival-SVM model for 1-year (A), 3-year (B), and 5-year (C) overall survival across the GSE87371, GSE10846, and NCICCR cohorts. (D–F) Calibration plots comparing the predicted versus observed survival probabilities at 1, 3, and 5 years for the GSE87371 (D), GSE10846 (E), and NCICCR (F) cohorts.*


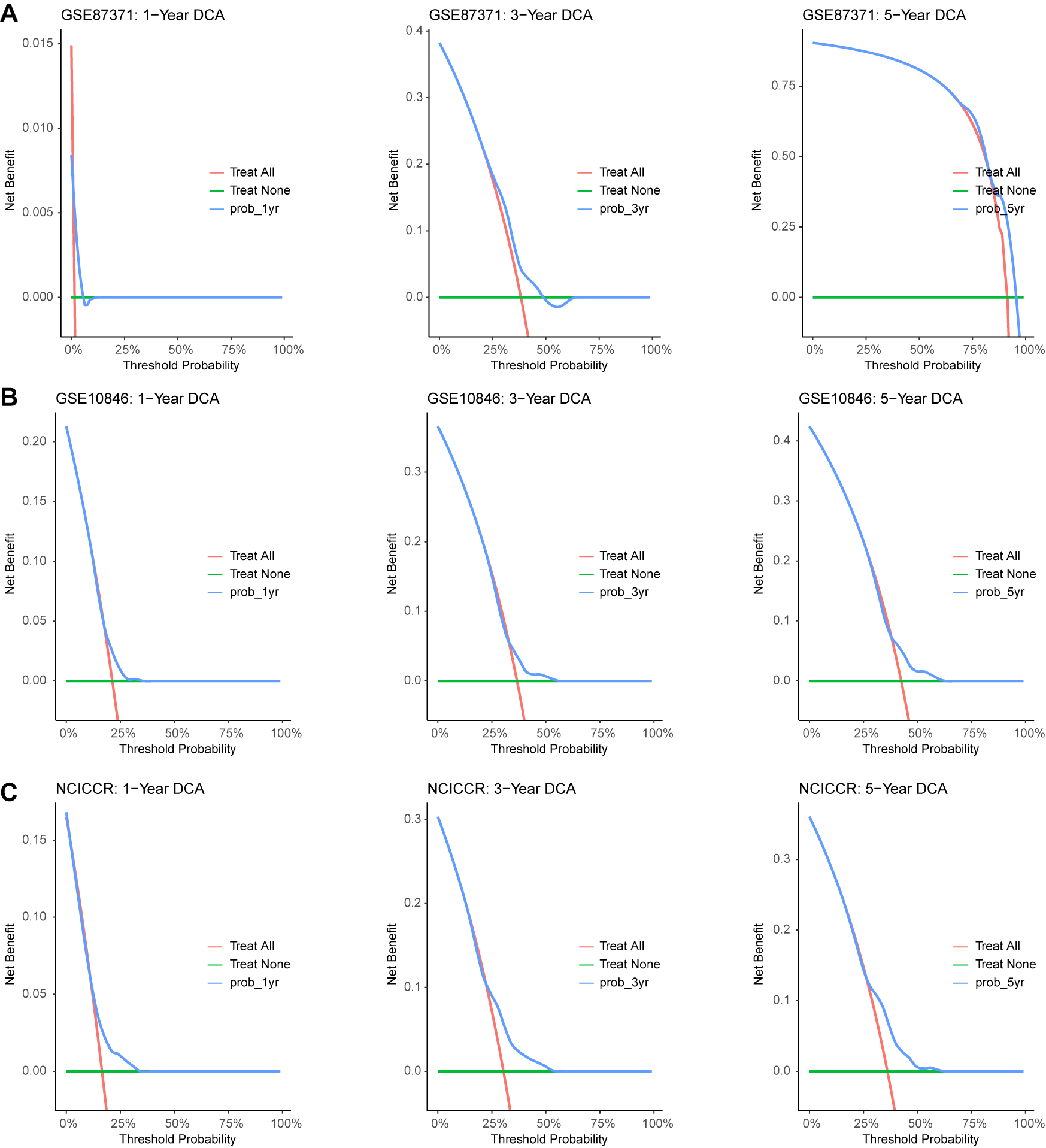


***Fig. S9.*** *Decision curve analysis (DCA) assessing the clinical utility of the prognostic signature. (A–C) DCA plots for 1-year, 3-year, and 5-year overall survival predictions in the GSE87371 (A), GSE10846 (B), and NCICCR (C) cohorts.*

**Table S1.** Primers for RT-qPCR.

| Gene | Forward Primer (5'→3') | Reverse Primer (5'→3') |
| --- | --- | --- |
| *TNFα* | CTCATGCACCACCATCAAGG | ACCTGACCACTCTCCCTTTG |
| *IL6* | ACTCACCTCTTCAGAACGAATTG | CCATCTTTGGAAGGTTCAGGTTG |
| *ARG1* | CAGTCGTGGGAGGTCTGACATACA | GCAAGTCCGAAACAAGCCAAGGT |
| *IL10* | GCCGTGGAGCAGGTGAAGAATG | GAGTCGCCACCCTGATGTCTCA |
| *GAPDH* | TGCTGAGTATGTCGTGGAGTC | GGATGCAGGGATGATGTTCT |

**Table S2.** Univariate Cox regression analysis of 132 candidate genes identified by the intersection of DEGs and the WGCNA steelblue module.

| Gene | GSE87371 | | | | GSE10846 | | | |
| --- | --- | --- | --- | --- | --- | --- | --- | --- |
|  | HR | HR.95L | HR.95H | p value | HR | HR.95L | HR.95H | *p value* |
| *ABI3BP* | 0.829593361 | 0.427312948 | 1.610588087 | 0.580997466 | 0.932799431 | 0.846648445 | 1.027716739 | 0.159426819 |
| *ACTN1* | 1.074529984 | 0.927121372 | 1.245375978 | 0.339664884 | 0.720650543 | 0.616116663 | 0.84292024 | 4.18396E-05 |
| *ADGRF5* | 1.074648212 | 0.940105893 | 1.22844542 | 0.291453738 | 0.853219494 | 0.713796501 | 1.019875419 | 0.081199142 |
| *ADGRL4* | 1.097444122 | 0.949351179 | 1.268638653 | 0.208678929 | 0.861224779 | 0.764033517 | 0.970779559 | 0.014470354 |
| *AMOTL2* | 1.083548151 | 0.904420932 | 1.298152833 | 0.384121282 | 0.855247866 | 0.734441567 | 0.995925267 | 0.044165587 |
| *ANO7* | 1.267829977 | 0.468261674 | 3.432680784 | 0.640525402 | 1.107199573 | 0.945035902 | 1.29718976 | 0.207557079 |
| *APOC1* | 1.253256061 | 1.075088445 | 1.460950271 | 0.003909259 | 0.73338356 | 0.631380806 | 0.851865373 | 4.94473E-05 |
| *APOE* | 1.192617049 | 1.042393946 | 1.364489338 | 0.010334933 | 0.920050028 | 0.794059579 | 1.066030908 | 0.267440606 |
| *ARMCX1* | 1.242870316 | 1.048117732 | 1.473810217 | 0.012404263 | 0.699464516 | 0.584046555 | 0.837691114 | 0.000102398 |
| *C1orf54* | 1.372900946 | 1.124948792 | 1.675504717 | 0.001817918 | 0.636320588 | 0.521272924 | 0.776759874 | 8.88266E-06 |
| *C1QA* | 1.107102639 | 1.015045785 | 1.207508341 | 0.021611829 | 1.207984382 | 1.116764405 | 1.306655423 | 2.39738E-06 |
| *C1QC* | 1.080643734 | 0.990469274 | 1.179027873 | 0.081062681 | 1.246374772 | 1.141628405 | 1.360731799 | 8.77303E-07 |
| *C1R* | 0.997341328 | 0.879734978 | 1.130669747 | 0.966829073 | 0.885586018 | 0.76010109 | 1.031787226 | 0.119097959 |
| *C1S* | 1.14243135 | 0.972962588 | 1.341417858 | 0.104079369 | 0.890664509 | 0.828149505 | 0.957898619 | 0.001818323 |
| *C3* | 0.966589342 | 0.878003187 | 1.0641134 | 0.488381286 | 0.812654931 | 0.745782753 | 0.885523344 | 2.1922E-06 |
| *CCL19* | 1.085219515 | 0.997232657 | 1.180969544 | 0.057996077 | 0.923752672 | 0.843613244 | 1.011504983 | 0.086729951 |
| *CD14* | 1.162539679 | 1.043259135 | 1.295458108 | 0.006397325 | 0.976445324 | 0.856896405 | 1.112672975 | 0.720553586 |
| *CD200* | 1.099004405 | 0.982043407 | 1.229895412 | 0.100103566 | 0.849078338 | 0.734040606 | 0.98214461 | 0.027629036 |
| *CD40LG* | 1.048786849 | 0.699550178 | 1.572373061 | 0.817665383 | 0.862987298 | 0.784016002 | 0.949913106 | 0.00261788 |
| *CD93* | 1.04218799 | 0.870514217 | 1.247717483 | 0.652737639 | 0.971661967 | 0.855178151 | 1.104012043 | 0.659049761 |
| *CDH11* | 1.055809112 | 0.925456659 | 1.204521973 | 0.419238923 | 0.771433897 | 0.696433012 | 0.854511845 | 6.59788E-07 |
| *CFH* | 1.094224115 | 0.989509584 | 1.210020028 | 0.079347492 | 0.870402681 | 0.76697644 | 0.987775879 | 0.031513376 |
| *CLIC2* | 1.324513641 | 1.112172673 | 1.577395693 | 0.001618698 | 0.752596482 | 0.657279098 | 0.861736615 | 3.89446E-05 |
| *CLU* | 1.094573983 | 0.469235009 | 2.553288187 | 0.834368894 | 1.12183065 | 0.990679123 | 1.270344735 | 0.069934534 |
| *CNRIP1* | 1.075053195 | 0.953644962 | 1.21191787 | 0.236547053 | 0.746139105 | 0.650539897 | 0.855786965 | 2.83712E-05 |
| *COL15A1* | 1.004835353 | 0.91791929 | 1.099981335 | 0.916770686 | 0.884915861 | 0.792776398 | 0.98776412 | 0.029300263 |
| *COL1A1* | 0.972696597 | 0.876450462 | 1.079511862 | 0.602542161 | 0.856044993 | 0.757913532 | 0.966882104 | 0.012345323 |
| *COL1A2* | 0.9873793 | 0.89097874 | 1.09421004 | 0.808538951 | 0.783370109 | 0.702937892 | 0.873005617 | 1.00072E-05 |
| *COL3A1* | 1.023671749 | 0.944746834 | 1.109190116 | 0.567649713 | 0.873348413 | 0.797781485 | 0.956073142 | 0.003358992 |
| *COL4A1* | 1.008949739 | 0.883756476 | 1.151877926 | 0.895131793 | 0.856060254 | 0.742235973 | 0.987339855 | 0.032761681 |
| *COL4A2* | 0.981710535 | 0.882696127 | 1.091831655 | 0.733634915 | 0.861614657 | 0.750384298 | 0.989332824 | 0.034683491 |
| *COL5A2* | 1.03087955 | 0.947640787 | 1.12142983 | 0.478951762 | 0.862873621 | 0.78499137 | 0.948482894 | 0.002244268 |
| *COL6A3* | 1.019351052 | 0.907861704 | 1.144531775 | 0.745700574 | 0.813146885 | 0.721309288 | 0.916677309 | 0.000717543 |
| *CPE* | 1.09705669 | 0.968928043 | 1.242128753 | 0.143787761 | 0.817549696 | 0.747498232 | 0.894166001 | 1.04574E-05 |
| *CRYGB* | 1.533864574 | 0.661927684 | 3.554376991 | 0.3184264 | 1.109783181 | 0.971405805 | 1.267872502 | 0.12527449 |
| *CSRP2* | 1.083316958 | 0.95585769 | 1.227772339 | 0.210181314 | 0.691089577 | 0.5966263 | 0.800509134 | 8.34556E-07 |
| *CTSD* | 1.180199213 | 1.005006371 | 1.385931694 | 0.043292659 | 1.030612147 | 0.902538488 | 1.176859946 | 0.656055276 |
| *CTSL* | 1.21340272 | 1.013115169 | 1.453286069 | 0.035594548 | 1.012997714 | 0.847536751 | 1.210760911 | 0.88713609 |
| *CXCL10* | 1.191540169 | 1.063866041 | 1.334536416 | 0.002440868 | 1.007527181 | 0.911163889 | 1.114081707 | 0.88376309 |
| *CXCL12* | 1.046820934 | 0.951137594 | 1.152129907 | 0.349466237 | 1.155241517 | 1.005936569 | 1.326706876 | 0.040974053 |
| *CXCL14* | 0.999712436 | 0.94544738 | 1.057092098 | 0.991941212 | 0.878032269 | 0.825356313 | 0.934070114 | 3.77824E-05 |
| *CXCL9* | 1.227049742 | 1.074743064 | 1.400940485 | 0.002478465 | 0.965383482 | 0.87179988 | 1.069012842 | 0.498290117 |
| *DCLK1* | 1.0334014 | 0.893772299 | 1.194843984 | 0.657316717 | 0.805897782 | 0.69586511 | 0.933329213 | 0.003962177 |
| *DCN* | 1.069924639 | 0.997372501 | 1.147754456 | 0.059223775 | 0.841317419 | 0.767180154 | 0.922619017 | 0.000241452 |
| *DPT* | 1.024650091 | 0.928131881 | 1.131205413 | 0.629505042 | 0.884679669 | 0.815206209 | 0.960073792 | 0.003320245 |
| *EFEMP1* | 1.052538116 | 0.94364254 | 1.174000151 | 0.358129926 | 0.822975749 | 0.73036511 | 0.927329461 | 0.001380922 |
| *ENPP2* | 1.137351854 | 0.962332515 | 1.344201946 | 0.131143114 | 0.767077069 | 0.669343121 | 0.879081613 | 0.00013711 |
| *EPB41L3* | 1.20042227 | 1.039436118 | 1.386341692 | 0.012903262 | 1.151459876 | 1.00466785 | 1.319699687 | 0.042673222 |
| *FBN1* | 0.98404223 | 0.896480226 | 1.080156687 | 0.735122204 | 0.796174317 | 0.727367704 | 0.87148981 | 7.70586E-07 |
| *FCER1G* | 1.197689088 | 1.070555627 | 1.339920239 | 0.001628489 | 1.065413235 | 0.933433848 | 1.216053353 | 0.34770013 |
| *FERMT2* | 1.155981799 | 0.994649123 | 1.343482731 | 0.058757098 | 0.855679232 | 0.771010506 | 0.949645877 | 0.00336955 |
| *FMOD* | 0.973098109 | 0.823557548 | 1.149792061 | 0.748711785 | 0.664818404 | 0.544333146 | 0.811972436 | 6.29042E-05 |
| *FN1* | 1.022946746 | 0.925260255 | 1.130946713 | 0.657739266 | 0.982853012 | 0.846417406 | 1.141280929 | 0.820556474 |
| *FRMD6* | 1.068193096 | 0.957497561 | 1.191686053 | 0.237262327 | 0.779076279 | 0.704652388 | 0.861360664 | 1.09764E-06 |
| *FSTL1* | 1.073389432 | 0.950157134 | 1.21260456 | 0.255021087 | 0.732953601 | 0.628592653 | 0.854640884 | 7.36211E-05 |
| *GJA1* | 1.047795122 | 0.927662344 | 1.18348516 | 0.452388243 | 0.745235129 | 0.66637488 | 0.833427871 | 2.56491E-07 |
| *GNAQ* | 1.204221893 | 1.020292823 | 1.421308015 | 0.027982662 | 0.742207442 | 0.609032582 | 0.904503147 | 0.003129497 |
| *GNLY* | 1.070960287 | 0.993737489 | 1.154184026 | 0.072584176 | 0.995423285 | 0.916132135 | 1.081577077 | 0.913747411 |
| *GPNMB* | 1.160124477 | 0.997122293 | 1.349773053 | 0.054522903 | 0.772854529 | 0.692197998 | 0.862909347 | 4.6074E-06 |
| *GPX3* | 1.054516577 | 0.959183284 | 1.159325053 | 0.272213313 | 1.182255838 | 1.073531667 | 1.301991279 | 0.000670139 |
| *GPX8* | 1.089983915 | 0.997136679 | 1.191476515 | 0.057849212 | 0.734617975 | 0.640071113 | 0.843130644 | 1.14698E-05 |
| *GXYLT2* | 1.058566539 | 0.956979206 | 1.17093779 | 0.268859048 | 0.715143211 | 0.628710447 | 0.81345843 | 3.37145E-07 |
| *HTRA1* | 1.034605956 | 0.93966974 | 1.139133716 | 0.488441643 | 0.707041841 | 0.601646584 | 0.830900031 | 2.56247E-05 |
| *ID1* | 1.110681543 | 0.981058009 | 1.257431751 | 0.097331714 | 1.075956197 | 0.900595445 | 1.285462575 | 0.419938524 |
| *IDO1* | 1.111400192 | 1.024352471 | 1.20584508 | 0.011143544 | 0.938488517 | 0.834963122 | 1.054849817 | 0.287080466 |
| *IFI27* | 1.163317182 | 1.058566801 | 1.278433127 | 0.001676969 | 1.086199919 | 0.968932761 | 1.217659586 | 0.156036761 |
| *IFITM1* | 1.295661604 | 1.082393003 | 1.550951445 | 0.004760604 | 0.963101006 | 0.80782839 | 1.148218556 | 0.675112558 |
| *IGFBP3* | 0.944973789 | 0.843995745 | 1.058033133 | 0.326297577 | 0.769430716 | 0.699698174 | 0.846112865 | 6.39383E-08 |
| *IGFBP7* | 1.139934386 | 0.92379905 | 1.406637519 | 0.22207646 | 0.669179072 | 0.573075293 | 0.781399294 | 3.80666E-07 |
| *IL18* | 1.238608401 | 1.040590452 | 1.474307946 | 0.016056416 | 0.741411018 | 0.655376855 | 0.838739259 | 1.99106E-06 |
| *IL1R1* | 1.198737118 | 1.039176478 | 1.38279754 | 0.012873232 | 0.759460858 | 0.654777786 | 0.880880213 | 0.00027682 |
| *IL4I1* | 1.189786568 | 1.052415441 | 1.345088661 | 0.005501243 | 0.926893098 | 0.822845488 | 1.044097376 | 0.211430215 |
| *ISLR* | 1.175450488 | 0.960566868 | 1.438404652 | 0.116559793 | 0.903309426 | 0.806105997 | 1.012234026 | 0.080009823 |
| *KDR* | 1.08294992 | 0.846746606 | 1.385043083 | 0.525560881 | 0.806154761 | 0.667226457 | 0.974010384 | 0.025559889 |
| *LAMB1* | 1.00341224 | 0.876645866 | 1.14850952 | 0.960573457 | 0.830875303 | 0.739290226 | 0.933806162 | 0.001875264 |
| *LGALS2* | 1.088470556 | 1.007673265 | 1.175746338 | 0.031223906 | 0.857451747 | 0.798095333 | 0.92122265 | 2.64806E-05 |
| *LGALS3BP* | 1.094587018 | 0.956428636 | 1.252702707 | 0.18923844 | 0.968926943 | 0.831914713 | 1.128504407 | 0.684887772 |
| *LINC01208* | 0.954242922 | 0.466531146 | 1.951808711 | 0.897924617 | 1.145357277 | 0.954121271 | 1.374923011 | 0.145368438 |
| *LOXL1* | 1.100543285 | 0.954759021 | 1.26858767 | 0.18636564 | 0.72920094 | 0.622748336 | 0.853850552 | 8.7702E-05 |
| *LPAR1* | 1.103307784 | 0.985048903 | 1.235764096 | 0.08921559 | 0.777420674 | 0.675478106 | 0.894748325 | 0.000446925 |
| *LUM* | 1.100939075 | 0.97901539 | 1.238046775 | 0.108314147 | 0.793201324 | 0.723657897 | 0.869427866 | 7.47249E-07 |
| *MAFB* | 1.170707816 | 0.984460193 | 1.392191173 | 0.074618234 | 0.948432842 | 0.801784527 | 1.121903486 | 0.53672139 |
| *MGP* | 1.04429487 | 0.954326404 | 1.142745051 | 0.345724383 | 0.903678431 | 0.815110112 | 1.001870415 | 0.054297282 |
| *MXRA5* | 1.034529416 | 0.962686418 | 1.111733885 | 0.355268964 | 0.806187434 | 0.730949828 | 0.889169343 | 1.63286E-05 |
| *MYOF* | 1.10700164 | 0.999994533 | 1.225459332 | 0.050012323 | 0.934622383 | 0.820792189 | 1.064238928 | 0.307551816 |
| *NPL* | 1.23734622 | 1.024657032 | 1.494183537 | 0.026892039 | 0.807471133 | 0.662374039 | 0.984352636 | 0.034343257 |
| *NR1H3* | 1.207613307 | 1.064657098 | 1.369764877 | 0.003339805 | 0.935275556 | 0.794815221 | 1.100558146 | 0.420286895 |
| *NUPR1* | 1.141072801 | 1.042490829 | 1.248977067 | 0.004201636 | 0.852470014 | 0.757314223 | 0.959582038 | 0.008213576 |
| *OLFML2B* | 1.090917052 | 0.977165888 | 1.217909904 | 0.121422273 | 0.951033728 | 0.823746181 | 1.097990101 | 0.493450139 |
| *OLFML3* | 1.077904472 | 0.973276293 | 1.193780286 | 0.149863457 | 0.851562792 | 0.753620208 | 0.962234268 | 0.009951803 |
| *PAPSS2* | 1.028364795 | 0.884983527 | 1.194976087 | 0.715046267 | 0.741929725 | 0.649137073 | 0.847986874 | 1.19349E-05 |
| *PLOD2* | 1.054508146 | 0.974849372 | 1.140676152 | 0.18538427 | 0.757182586 | 0.682106226 | 0.840522263 | 1.78015E-07 |
| *PLS3* | 1.142696574 | 0.992818724 | 1.315200276 | 0.062957086 | 0.738078248 | 0.647316176 | 0.841566332 | 5.7206E-06 |
| *PLTP* | 1.107564745 | 0.988557907 | 1.240898136 | 0.078147316 | 1.343318038 | 1.202772026 | 1.500287096 | 1.65557E-07 |
| *PPIC* | 1.072309737 | 0.942338961 | 1.22020655 | 0.289576879 | 0.71862714 | 0.624129004 | 0.827433052 | 4.36174E-06 |
| *PRRX1* | 0.995193361 | 0.842884224 | 1.175024751 | 0.954663025 | 0.696268944 | 0.572856653 | 0.846268328 | 0.000276028 |
| *PRTFDC1* | 1.077609313 | 0.936594209 | 1.239855874 | 0.296233678 | 0.76010879 | 0.664804462 | 0.869075655 | 5.99714E-05 |
| *PTGDS* | 1.084812336 | 0.982194105 | 1.198151973 | 0.108359664 | 0.809718548 | 0.727379467 | 0.901378383 | 0.000114489 |
| *RAI14* | 1.056690798 | 0.925563781 | 1.206394918 | 0.414667667 | 0.857361068 | 0.743209398 | 0.989045622 | 0.034767244 |
| *RARRES1* | 1.083797331 | 0.971144171 | 1.209518308 | 0.15069848 | 0.806960933 | 0.73036772 | 0.891586429 | 2.4952E-05 |
| *RARRES2* | 1.129389994 | 1.016575975 | 1.254723492 | 0.023442363 | 0.69132788 | 0.618070683 | 0.773267931 | 1.05263E-10 |
| *RASSF4* | 2.068003481 | 1.34249438 | 3.185591285 | 0.000980503 | 1.076059426 | 0.952185454 | 1.216048705 | 0.240084156 |
| *RND3* | 1.143793982 | 1.025336576 | 1.275936804 | 0.016017532 | 0.827375329 | 0.702573353 | 0.974346568 | 0.023117781 |
| *RSPO3* | 1.072042239 | 0.947120296 | 1.213440961 | 0.27111516 | 0.761307329 | 0.670131863 | 0.864887764 | 2.78662E-05 |
| *RTN1* | 1.142624036 | 1.010821447 | 1.291612571 | 0.032999959 | 0.738128749 | 0.649706763 | 0.838584545 | 3.10068E-06 |
| *S100A4* | 1.027900947 | 0.86362921 | 1.223418967 | 0.756755756 | 0.952150791 | 0.823305775 | 1.101159687 | 0.508638357 |
| *S1PR3* | 1.044889073 | 0.932198726 | 1.171202174 | 0.450759912 | 0.837233773 | 0.70850679 | 0.989348869 | 0.037007451 |
| *SASH1* | 1.25662012 | 1.035619732 | 1.524781807 | 0.02063396 | 1.082660264 | 0.951721454 | 1.231613769 | 0.227206703 |
| *SDC4* | 1.242319106 | 1.080028077 | 1.428996888 | 0.00238298 | 0.848483816 | 0.717519373 | 1.0033524 | 0.054752196 |
| *SERPING1* | 1.074210703 | 0.972046425 | 1.187112678 | 0.160338153 | 1.024534744 | 0.903300199 | 1.162040528 | 0.706009531 |
| *SLAMF8* | 1.17172189 | 1.051573942 | 1.305597383 | 0.004091625 | 0.912205151 | 0.778911452 | 1.068309159 | 0.254236686 |
| *SNAI2* | 1.058545468 | 0.944733137 | 1.186068812 | 0.326912259 | 0.846478928 | 0.750808955 | 0.95433941 | 0.006455072 |
| *SPARC* | 1.02202061 | 0.859964901 | 1.214614838 | 0.804693684 | 0.710243693 | 0.602579841 | 0.837144008 | 4.51618E-05 |
| *SPARCL1* | 1.17033906 | 0.979970545 | 1.397688453 | 0.082460301 | 0.868822364 | 0.758124349 | 0.99568402 | 0.043159295 |
| *SPATA4* | 1.136946681 | 0.369090969 | 3.502247042 | 0.823074725 | 1.07219823 | 0.947449359 | 1.21337255 | 0.269333088 |
| *TDO2* | 1.11883055 | 1.025880106 | 1.22020282 | 0.0111694 | 0.773038828 | 0.68990158 | 0.866194609 | 9.23358E-06 |
| *TECTB* | 0.56338858 | 0.214269992 | 1.481339917 | 0.244708175 | 1.071219501 | 0.9346232 | 1.227779516 | 0.322907055 |
| *TFPI* | 1.115333102 | 0.972544913 | 1.279085326 | 0.118366332 | 0.962866415 | 0.858623982 | 1.079764546 | 0.517459046 |
| *TGFBI* | 1.037979086 | 0.932007593 | 1.155999792 | 0.497506101 | 0.792474189 | 0.702010928 | 0.894594822 | 0.000169213 |
| *THY1* | 0.971451706 | 0.846748431 | 1.114520421 | 0.679464547 | 0.767591515 | 0.655708457 | 0.898565098 | 0.000999743 |
| *TIMP3* | 1.037144922 | 0.954971351 | 1.126389381 | 0.386499039 | 0.878333824 | 0.793356564 | 0.972413088 | 0.012461303 |
| *TLR8* | 1.144583161 | 1.01102896 | 1.295779512 | 0.032905479 | 0.971291678 | 0.901412353 | 1.046588191 | 0.44448861 |
| *TMEM119* | 1.026227444 | 0.935276185 | 1.12602329 | 0.584533024 | 0.806105403 | 0.734500498 | 0.88469092 | 5.58998E-06 |
| *TMEM163* | 1.022288183 | 0.922192999 | 1.133247737 | 0.675011744 | 0.77853279 | 0.686894628 | 0.882396339 | 8.92465E-05 |
| *TMEM176A* | 1.12604535 | 0.968856605 | 1.308736631 | 0.121737088 | 0.862928364 | 0.712679049 | 1.044853728 | 0.130935168 |
| *TMEM176B* | 1.17627479 | 1.022373519 | 1.35334333 | 0.023253941 | 0.942779945 | 0.791016324 | 1.123660787 | 0.510547883 |
| *TMEM47* | 1.066514397 | 0.942037816 | 1.207438746 | 0.30916128 | 0.828733128 | 0.769056325 | 0.893040699 | 8.36227E-07 |
| *TNFRSF1A* | 1.21462658 | 1.030985841 | 1.430977683 | 0.02008107 | 1.197214599 | 1.012935159 | 1.415019297 | 0.034800661 |
| *VCAM1* | 1.092245641 | 0.922642106 | 1.293026335 | 0.30544547 | 0.826607819 | 0.701107943 | 0.97457245 | 0.023418995 |
| *VCAN* | 1.051476158 | 0.96806799 | 1.142070725 | 0.23390682 | 0.854107657 | 0.776155495 | 0.939888843 | 0.001239833 |
| *WHAMMP2* | 1.475073278 | 0.8689051 | 2.504118316 | 0.149994019 | 1.33144071 | 1.127961438 | 1.571626746 | 0.000717095 |
| *YAP1* | 1.086326436 | 0.929678816 | 1.269368631 | 0.297321036 | 0.836451801 | 0.75697002 | 0.924279162 | 0.000455487 |

**Table S3.** Marker genes of TNFRSF1A^+^ TAMs.

| Gene | avg_log_2_FC | pct.1 | pct.2 | *p_val_adj* |
| --- | --- | --- | --- | --- |
| ADAP2 | 0.265864241 | 0.629 | 0.389 | 1.38498E-05 |
| ANKRD22 | 0.427515676 | 0.747 | 0.491 | 1.90249E-07 |
| ATP5MC3 | 0.27888469 | 0.981 | 0.82 | 2.22173E-06 |
| AUP1 | 0.253207165 | 0.85 | 0.575 | 2.0656E-06 |
| CALHM6 | 0.434918759 | 0.954 | 0.784 | 2.74613E-07 |
| CALR | 0.34898623 | 0.986 | 0.826 | 1.78437E-06 |
| *CANX* | 0.27486567 | 0.91 | 0.671 | 1.19858E-07 |
| *CCL18* | 0.292668093 | 0.545 | 0.216 | 1.63139E-10 |
| *CD1E* | 0.281225493 | 0.243 | 0.144 | 0.008723301 |
| *CD300A* | 0.279716987 | 0.73 | 0.449 | 1.29604E-06 |
| *CD44* | 0.282945236 | 0.932 | 0.796 | 0.000879546 |
| *CDKN1A* | 0.285739549 | 0.594 | 0.431 | 0.002844644 |
| *CFB* | 0.263855252 | 0.362 | 0.18 | 3.98605E-05 |
| *CIITA* | 0.339038767 | 0.681 | 0.353 | 6.197E-09 |
| *CLEC10A* | 0.525997891 | 0.728 | 0.545 | 0.000526891 |
| *CSF1R* | 0.314439826 | 0.823 | 0.593 | 5.01689E-06 |
| *CXCL10* | 0.412673522 | 0.578 | 0.455 | 0.001535576 |
| *CYB5R3* | 0.273055144 | 0.763 | 0.443 | 3.73724E-07 |
| *DPYD* | 0.334717657 | 0.736 | 0.401 | 2.72107E-09 |
| *DRAM1* | 0.272621283 | 0.676 | 0.539 | 0.005864158 |
| *GBP4* | 0.330340276 | 0.733 | 0.533 | 0.000150188 |
| *GCH1* | 0.285223226 | 0.616 | 0.413 | 0.000102689 |
| *HRH2* | 0.324830248 | 0.599 | 0.335 | 5.7251E-07 |
| *HSP90B1* | 0.280680366 | 0.965 | 0.76 | 9.64499E-06 |
| *ICAM1* | 0.350258551 | 0.85 | 0.623 | 2.23835E-06 |
| *IL1B* | 0.490773189 | 0.523 | 0.335 | 0.000199064 |
| *LILRB2* | 0.270089267 | 0.744 | 0.545 | 0.000404354 |
| *MGAT1* | 0.296028672 | 0.888 | 0.635 | 5.7153E-06 |
| *MRC1* | 0.295344365 | 0.346 | 0.21 | 0.002494105 |
| *MYD88* | 0.281806017 | 0.88 | 0.623 | 6.93829E-07 |
| *NOP10* | 0.367290825 | 0.627 | 0.287 | 2.19493E-11 |
| *NUCKS1* | 0.295837063 | 0.831 | 0.527 | 2.82837E-07 |
| *OTULINL* | 0.311379613 | 0.662 | 0.311 | 5.16717E-10 |
| *PPA1* | 0.257082973 | 0.975 | 0.802 | 7.3847E-06 |
| *PSTPIP2* | 0.297453454 | 0.662 | 0.443 | 6.01425E-06 |
| *PTPRC* | 0.277227391 | 0.967 | 0.814 | 3.15945E-05 |
| *RASSF4* | 0.255899069 | 0.872 | 0.635 | 3.4927E-05 |
| *RER1* | 0.258501587 | 0.853 | 0.551 | 2.02374E-06 |
| *RHOB* | 0.299583781 | 0.828 | 0.485 | 2.22377E-09 |
| *RNF213* | 0.299330609 | 0.853 | 0.659 | 3.62034E-05 |
| *SAMHD1* | 0.308693548 | 0.965 | 0.778 | 6.18778E-05 |
| *SFT2D2* | 0.26162797 | 0.725 | 0.431 | 4.51586E-07 |
| *SOCS3* | 0.386911312 | 0.583 | 0.287 | 1.20888E-07 |
| *TLR2* | 0.295366541 | 0.689 | 0.371 | 3.01652E-08 |
| *TNFRSF1A* | 1.334245801 | 1 | 0 | 4.42026E-79 |
| *TNFSF13B* | 0.306977157 | 0.695 | 0.347 | 3.5502E-09 |
| *TPR* | 0.253794432 | 0.749 | 0.413 | 2.02089E-08 |
| *ZEB2* | 0.359560936 | 0.864 | 0.545 | 3.17654E-09 |

**Table S4** Relative importance of the 7 genes within the prognostic signature based on "Survival-SVM" algorithm.

| Rank | Gene | Risk_Coefficient |
| --- | --- | --- |
| 1 | *CXCL10* | 12.83583861 |
| 2 | *ANKRD22* | 11.18934979 |
| 3 | *CLEC10A* | 9.689569576 |
| 4 | *CCL18* | 9.501008082 |
| 5 | *PSTPIP2* | 8.114736353 |
| 6 | *MGAT1* | 7.251266959 |
| 7 | *TNFRSF1A* | 1.467101071 |
